# Cause-specific mortality for 249 causes in Brazil and states during 1990–2015: a systematic analysis for the global burden of disease study 2015

**DOI:** 10.1186/s12963-017-0156-y

**Published:** 2017-11-22

**Authors:** Elisabeth B. França, Valéria Maria de Azeredo Passos, Deborah Carvalho Malta, Bruce B. Duncan, Antonio Luiz P. Ribeiro, Mark D. C. Guimarães, Daisy M.X. Abreu, Ana Maria N. Vasconcelos, Mariângela Carneiro, Renato Teixeira, Paulo Camargos, Ana Paula S. Melo, Bernardo L. Queiroz, Maria Inês Schmidt, Lenice Ishitani, Roberto Marini Ladeira, Otaliba L. Morais-Neto, Maria Tereza Bustamante-Teixeira, Maximiliano R. Guerra, Isabela Bensenor, Paulo Lotufo, Meghan Mooney, Mohsen Naghavi

**Affiliations:** 1 Universidade Federal de Minas Gerais, Faculdade de Medicina, Programa de Pós-graduação em Saúde Pública, Av. Prof. Alfredo Balena, 190, Belo Horizonte, 30130-100 Brazil; 2 Universidade Federal de Minas Gerais, School of Medicine, Av. Alfredo Balena, 190., Belo Horizonte, 30130-100 Brazil; 3 Universidade Federal de Minas Gerais, Escola de Enfermagem, Departamento de Enfermagem Materno Infantil, Av. Prof. Alfredo Balena, 190, Belo Horizonte, 30130-100 Brazil; 4 Universidade Federal do Rio Grande do Sul, Programa de Pós-Graduação em Epidemiologia, R. Ramiro Barcelos 2600/414, Porto Alegre, 90035-003 Brazil; 5 Universidade Federal de Minas Gerais, Faculdade de Medicina, Nescon, Av. Prof. Alfredo Balena, 190, Belo Horizonte, 30130-100 Brazil; 6 Universidade de Brasília, Programa de Pós-graduação em Desenvolvimento, Sociedade e Cooperação Internacional, Asa Norte, Brasília, 70910-900 Brazil; 7 Universidade de Federal de Minas Gerais, Instituto de Ciências Biológicas, Departamento de Parasitologia. Avenida Antônio Carlos, 6670, Belo Horizonte, MG 31270-901 Brazil; 8 Universidade Federal de São João Del Rei, Faculdade de Medicina. Praça Frei Orlando, 170, Centro, São João del-Rei, 36307-352 Brazil; 9 Universidade Federal de Minas Gerais, Faculdade de Ciências Econômicas, Departamento de Demografia, Av. Antônio Carlos, 6670, Belo Horizonte, 31270-901 Brazil; 10 Universidade Federal de Minas Gerais, Grupo de Pesquisas em Epidemiologia e Avaliação em Saúde-GPEAS, Av. Alfredo Balena, 190, Belo Horizonte, 30130-100 Brazil; 11 Fundação Hospitalar do Estado de Minas Gerais, Alameda Alvaro Celso 100/231, Belo Horizonte, 30150-260 Brazil; 12 Universidade Federal de Goiás, Departamento de Saúde Coletiva. Instituto de Patologia Tropical e Saúde Pública. Rua 235, S/N, Setor Universitário, Goiânia, Goiás, 74605050 Brazil; 13 Universidade Federal de Juiz de Fora, Programa de Pós-graduação em Saúde Coletiva, Campus Universitario da UFJF, Rua José Lourenço Kelmer, S/n, Martelos, Juiz de Fora, 36036-330 Brazil; 14 Universidade de São Paulo. Centro de Pesquisa Clínica e Epidemiológica, Hospital Universitário, Av. Lineu Prestes, 2565 / 3° andar, São Paulo, 05508-000 Brazil; 15 Institute for Health Metrics and Evaluation, 2301 5th Avenue, Suite 600, Box 358210, Seattle, WA 98121 USA

**Keywords:** Mortality, Causes of death, Global burden of disease, Brazil

## Abstract

**Background:**

Reliable data on cause of death (COD) are fundamental for planning and resource allocation priorities. We used GBD 2015 estimates to examine levels and trends for the leading causes of death in Brazil from 1990 to 2015.

**Methods:**

We describe the main analytical approaches focused on both overall and specific causes of death for Brazil and Brazilian states.

**Results:**

There was an overall improvement in life expectancy at birth from 1990 to 2015, but with important heterogeneity among states. Reduced mortality due to diarrhea, lower respiratory infections, and other infectious diseases contributed the most for increasing life expectancy in most states from the North and Northeast regions. Reduced mortality due to cardiovascular diseases was the highest contributor in the South, Southeast, and Center West regions. However, among men, intentional injuries reduced life expectancy in 17 out of 27 states. Although age-standardized rates due to ischemic heart disease (IHD) and cerebrovascular disease declined over time, these remained the leading CODs in the country and states. In contrast, leading causes of premature mortality changed substantially - e.g., diarrheal diseases moved from 1st to 13th and then the 36th position in 1990, 2005, and 2015, respectively, while violence moved from 7th to 1st and to 2nd. Overall, the total age-standardized years of life lost (YLL) rate was reduced from 1990 to 2015, bringing the burden of premature deaths closer to expected rates given the country’s Socio-demographic Index (SDI). In 1990, IHD, stroke, diarrhea, neonatal preterm birth complications, road injury, and violence had ratios higher than the expected, while in 2015 only violence was higher, overall and in all states, according to the SDI.

**Conclusions:**

A widespread reduction of mortality levels occurred in Brazil from 1990 to 2015, particularly among children under 5 years old. Major shifts in mortality rates took place among communicable, maternal, neonatal, and nutritional disorders. The mortality profile has shifted to older ages with increases in non-communicable diseases as well as premature deaths due to violence. Policymakers should address health interventions accordingly.

**Electronic supplementary material:**

The online version of this article (10.1186/s12963-017-0156-y) contains supplementary material, which is available to authorized users.

## Background

Brazil, a middle-income country with 8.5 million km^2^, is home to the world’s fifth largest population, around 200 million inhabitants, more than 80% of which live in urban areas [1]. It ranks among the ten largest economies in the world, and has shown important improvements in socioeconomic levels. In particular, during the last decade, 29 million people are no longer in poverty, and inequality has dropped significantly [2]. Despite this, given its 2014 Human Development Index value of 0.755, Brazil ranks 75th out of 188 countries [3], showing that many social challenges remain.

Since 1988, a national health system (the Unified Health System, or SUS) has been developed in order to provide universal and equitable health care to all citizens. Despite limitations and challenges inherent to the consolidation of this huge health system, ongoing SUS politics aim to guarantee free universal access to all domains of health care (primary, secondary, tertiary, and emergency care), universal coverage for vaccinations, prenatal care, access to medium and high complexity procedures such as hemodialysis, transplants, and cardiac surgery, as well as free access to essential drugs for NCDs (Alzheimer’s and Parkinson’s diseases, hypertension, diabetes, asthma, COPD), AIDS, and cancer [4]. The Family Health Strategy (FHS) was developed as an important innovation aiming to deliver community-based primary care. This program was scaled up from about 2000 teams providing service to 7 million people in 1998, and actively covering more than 100 million in 2013–2014 [4–6].

Within this scenario, policymakers need a better understanding of levels and trends in population health when planning and defining priorities for resource allocation. Cause-specific mortality is one of the fundamental metrics of population health. Thus, in 1975 the Mortality Information System (SIM in Portuguese) was conceived by the Ministry of Health to strengthen the information on cause of death data at the population level, complementing the work of the Brazilian Institute of Geography and Statistics (IBGE) in vital statistics. Though many actions have been taken to improve the quality of mortality data, over recent decades Brazil has still presented problems of completeness of registration and proportions of deaths classified to non-specific causes of death, with major differentials among geographic regions [7].

The Global Burden of Disease (GBD) study published in 1996 represented a paradigm shift from the traditional analysis of descriptive epidemiology of deaths and diseases [8]. One of the main advantages of the GBD approach is that consistent methods and standardized metrics are applied to make information for each condition comparable across time and space [9, 10]. The latest version of the study, GBD 2015, extends this approach to include disaggregated estimates for Brazilian states [11]. In the absence of this more adequate methodology, regional inequalities might remain hidden by the differences of data quality among subnational units, as areas with high burden of disease are often underrepresented on vital statistics [12].

Several studies have been published with data analysis of mortality risks in Brazil, but only more recently have a few works taken into account completeness of the SIM and adjusted for variation in death certificate misclassification, and those only reported mortality for specific diseases or specific areas [13–17]. Thus, to our knowledge, there have been no systematic studies on causes of death by Brazilian states, with correction of underregistration and unspecified codes of cause of death. In this study, we used the GBD 2015 study estimates to examine levels and time trends for 249 causes of death for all 26 Brazilian states and the Federal District in 1990 and 2015.

## Methods

### Overview of the methods used

This study is based on the GBD 2015 estimates of deaths and mortality rates, as already described elsewhere in greater detail [11]. Here, we summarize the main approach used and describe specific issues related to the estimation of all-cause mortality and causes of death for Brazilian states.

The estimation of all-cause mortality (“the mortality envelope”) is related to how well the event death was captured by the mortality information system. Thus, the first main component in COD analysis is the estimation of all-cause mortality and age-specific mortality for correcting undercounting, using a combination of model-life tables, death distribution methods, and regression techniques. The second step is the COD estimation which corrects issues in the ascertainment of the underlying cause, such as ill-defined codes or poorly specified causes of death, called garbage codes. GBD cause of death ensemble modeling (CODEm) combines the results of several different approaches to estimate cause-specific mortality, favoring those with best independent (out-of-sample) performance, i.e., holding out some fraction of the data from model building and then comparing the predictions to the data held out of the analysis [18]. For selected causes, other models were also used. The CodCorrect algorithm was then used to rescale the cause estimates in a hierarchical manner, and fitting the mortality envelope [11].

CODs have been reported using ICD-9 from 1990 to 1995, and ICD-10, adopted in the country since 1996. ICD 4-digit codes from SIM were mapped to the GBD cause list [7, 11]. This list places all causes within a four-level hierarchy. The first level divides the causes in three groups: communicable, maternal, neonatal, and nutritional disorders; non-communicable diseases (NCDs); and, injuries. The second level consists of 20 major CODs such as neonatal disorders, cardiovascular diseases, and transport injuries. The third level subdivides level 2 into types such as neonatal preterm birth complications, cerebrovascular disease, and road traffic injuries, and the fourth level further subdivides those types; for example, road traffic injuries in pedestrian, cyclist, motorcyclist, motor vehicle, and other road injuries [11].

In this paper, most results are reported at Level 3 of the GBD, and we focus on specific issues related to the analysis of top causes of death in Brazil, or causes with a higher percent of change in risks from 1990 to 2015. The units of analyses are the 27 Federative Units (26 states and the Federal District), herein simply named as states. To enhance interpretation, analyses are sometimes presented by grouping the 27 states into the five traditional Brazilian regions - North, Northeast, Southeast, South, and Center West:

1. North region: seven states (Acre, Amapá, Amazonas, Rondônia, Roraima, Tocantins) comprising 8.6% of Brazil’s total population in 2010;
2. Northeast: nine states (Alagoas, Bahia, Ceará, Maranhão, Paraíba, Pernambuco, Piauí, Rio Grande do Norte, Sergipe) - 27.7% of the total population;
3. Southeast: four states (Espírito Santo, Minas Gerais, Rio de Janeiro, São Paulo) -41.9% of the total population;
4. South: three states (Paraná, Rio Grande do Sul, Santa Catarina) - 14.3% of the total population;
5. Center West: the Federal District and three states (Goiás, Mato Grosso, Mato Grosso do Sul) - 7.6% of the total population [1].

### Estimating all-cause and age-specific deaths to correct for undercounting

Registration of deaths is a legal requirement in Brazil. However, completeness of vital registration data has varied across states during the past 25 years [7, 19]. To calculate the mortality envelope, i.e., the total number of deaths estimated for each age, sex, and year separately, sources of all-cause mortality data at the state levels in Brazil were systematically reviewed for the time period 1960–2015.

The main sources of data are the SIM, periodic national surveys, and censuses. Summary birth history from censuses (1960, 1970, 1980, 1991, 2000, 2010) and from surveys such as the Brazil National Household Sample Survey (PNAD in Portuguese) from 1992 to 2009, and also complete birth histories from the Demographic Health Survey (DHS 1986, 1996, and 2006) were used both as sources of data and to assess the completeness of under-5 death registration from SIM [11]. Under-5 mortality (ages 0–4) and adult mortality (ages 15–59) completeness are estimated separately. For adult mortality (45q15), the GBD 2015 study used the proposal of Murray and colleagues to estimate completeness of death counts [20]. Further details were already described [11].

### Cause of death estimation

Data on causes of death were provided by SIM. This system has been based on the international form of the death certificate (DC) since 1976. All deaths require certification of cause of death (COD) by a physician. For deaths that occur at home without physician certification, CODs are certified by the Death Investigation Service [*Serviço de Verificação de Óbitos (SVO*)], an official institution which performs autopsy in non-external deaths with unknown cause (home or hospital deaths), or by the civil registrar, in which case the COD is not recorded. For suspicious deaths, the DC is issued by a coroner from forensic institutes. Coding of death certificates in SIM is undertaken using an automated coding system [7].

Since 2006, the Ministry of Health has promoted the investigation by health services of ill-defined causes from chapter 18 ICD-10 (R codes) to re-assign an appropriate COD. Methods used to investigate those deaths include medical record review, analysis at the municipal level of record linkages between health information systems, autopsy results, and/or physician certified verbal autopsy. The latter are currently undertaken using the WHO long-form questionnaire [21].

Although the proportion of ill-defined R codes decreased notably after 2006, this proportion is still relatively high and presents important differentials across states and over time. To correct for these problems [21], we used the GBD method for the redistribution of garbage codes for the country and for subnational data [11]. A table in Additional file 1 presents the most important ill-defined R codes and other garbage codes by Brazilian states in 2014.

### Age-standardized death rates and years of life lost, and uncertainty intervals

The corrected number of deaths in each sex and five-year age group by state was used to produce age-specific mortality rates by applying population denominators. The number of years of life lost (YLLs) was calculated by multiplying numbers of deaths from each cause in each age-group by the reference life expectancy, considering the GBD 2015 standard life table. To produce age-standardized death rates and YLL age-standardized rates (/100,000 population) we used the world population as standard [11].

Each metric (e.g., death rates, YLLs) is reported with 95% uncertainty intervals (UIs), which include uncertainty from all sources and all modeling stages. Each quantity of interest is estimated 1000 separate times. Each estimate represents one draw. The uncertainty interval is the 2.5th and 97.5th centile of the draws [11].

### Estimation of the socio-demographic index (SDI)

To better understand a given country’s health indicators relative to those of other countries of similar socioeconomic level, a composite indicator, the SDI, has been created by the GBD study for each country/subnational region. Estimates of the SDI are based on three indicators: 1) lag dependent income per capita*,* 2) average educational attainment in the population over age 15, and 3) total fertility rate. Methods previously used to produce the Human Development Index (HDI) were applied to generate a scale weighting each indicator equally and rescaling each one with values varying from zero (the lowest value observed in the time period 1980 to 2015) to one (the highest value observed), and each country’s value of the three indicators is expressed upon that scale.

**Fig. 1:**
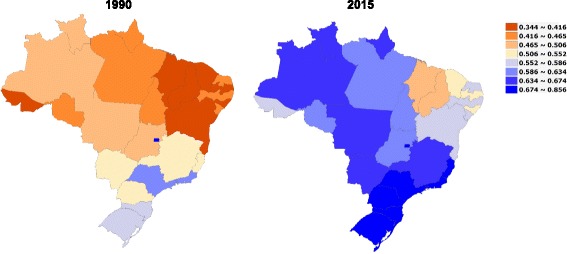
Socio-demographic Index (SDI) based on the GBD 2015 study in Brazilian states in 1990 and 2015. Legend

The SDI is then calculated as the geometric mean of the country’s values for the three indicators. The highest level of SDI can be interpreted as the highest observed lag income per capita and educational attainment, and the lowest fertility rate. Figure 1 presents the SDI for Brazilian states in 1990 and 2015.

Countries and subnational divisions were classified into SDI quintiles in 2015, based on the entire distribution of geography-years from 1980 to 2015, after excluding populations less than 1 million. Expected death rates by age-sex-cause were calculated for each SDI level. For example, expected age-standardized cause-specific YLL rates were computed on the basis of the age-sex-cause-specific predictions given the SDI level [11].

## Results

### Changes in age-sex-specific mortality rates and life expectancy by cause

**Fig. 2:**
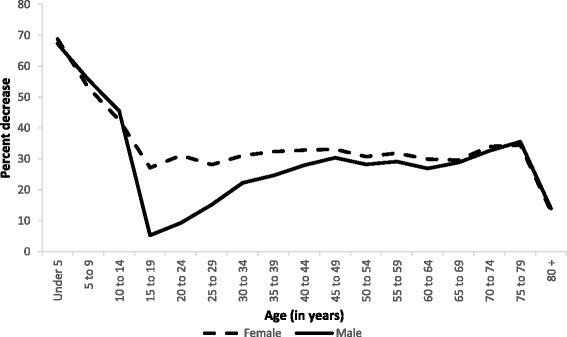
Percent decrease of age-specific mortality rates for females and males. Brazil, 1990 and 2015. Legend Female, Male

Age-sex-specific mortality rates in Brazil in 2015 compared to 1990 had the highest decrease for under--5 child deaths, for both sexes combined, and the lowest decrease for age 15–19 in males. This age group also presented the largest difference in percent change of risk between males and females. For all adult age groups, except for 75 and over, male individuals showed a more modest reduction in mortality rates than females (Fig. 2).

**Fig. 3:**
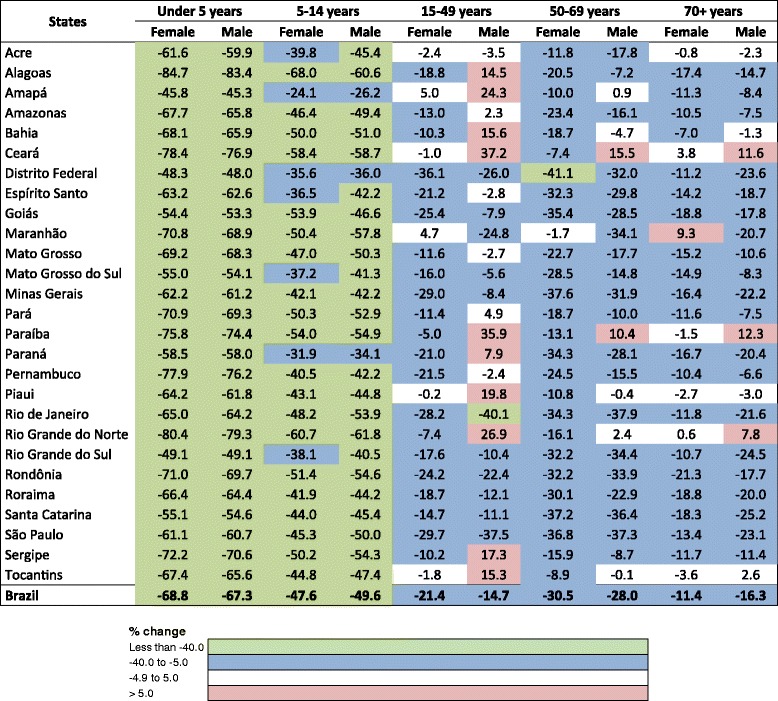
Percent change (%) of age-specific mortality rates for females and males by Brazilian states, 1990 and 2015. Legend

Figure 3 shows the percent changes in age-standardized mortality rates from 1990 to 2015 in Brazilian states. Major declines of mortality rates for those under 15 years can be observed uniformly across states and in both sexes, but lesser declines and a more variable pattern in the risk of death was seen among adults and elderly persons. Among young adult males (15–49 years), the risk of death increased in 12 of 27 states, mainly in those located in the North and the Northeast regions. On the other hand, São Paulo and Rio de Janeiro states presented an important decrease of this risk, around 40%. Male and female adults from 50 to 69 years had different decreases in the risk of death, greater than 25% in states from the South and Southeast regions, and also in Federal District, Goiás, and Rondônia. However, the risk of death increased for males in Ceará (15.5%) and Paraíba (10.4%). Variations in age-standardized mortality rates were also observed among elderly men, varying from a 25.2% decrease in Santa Catarina state to 12.3% increase in Paraíba state.

**Fig. 4:**
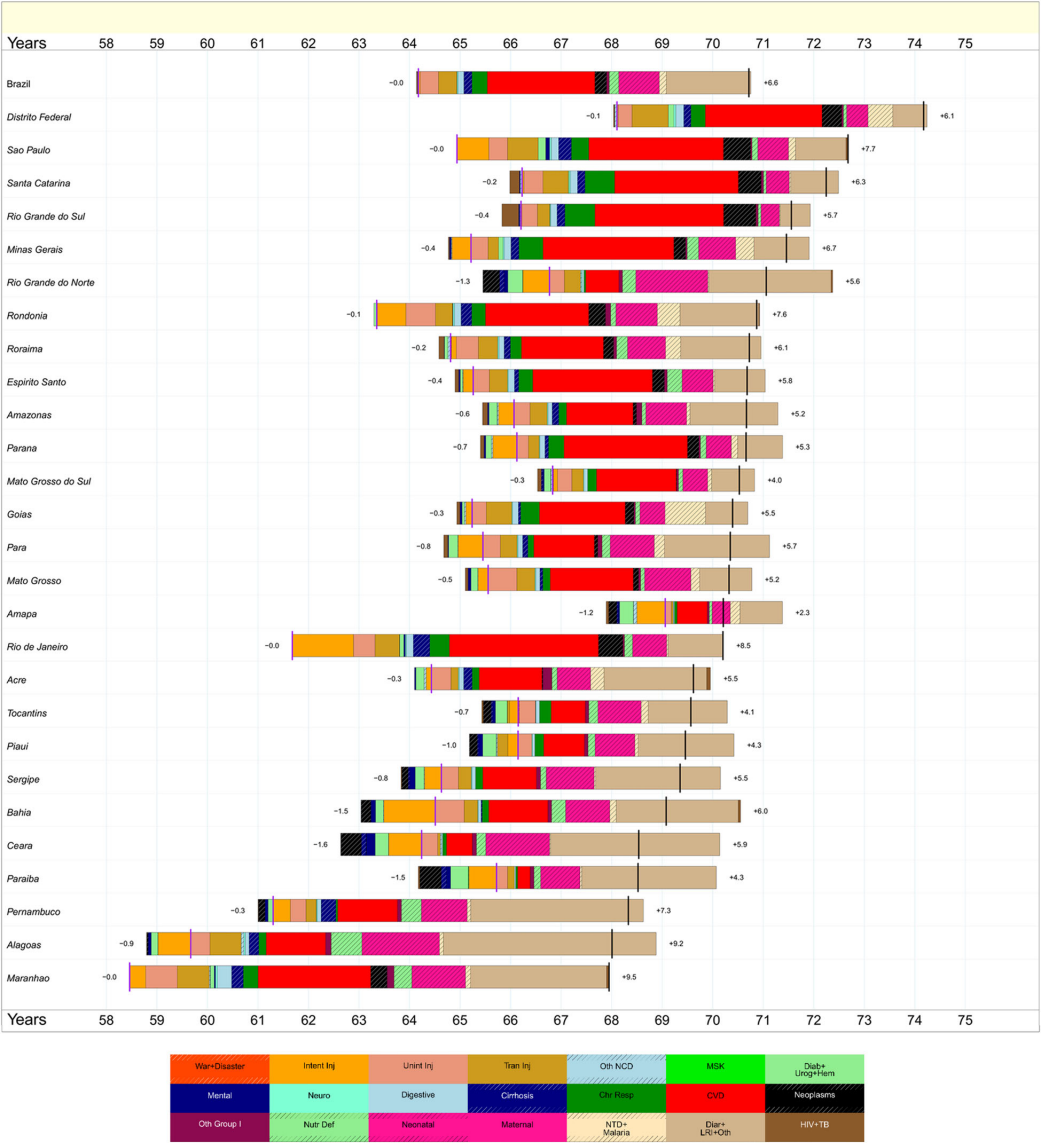
Attribution of changes in life expectancy to changes in major groups of causes of death for males in Brazil’s states, 1990 to 2015. Legend

**Fig. 5:**
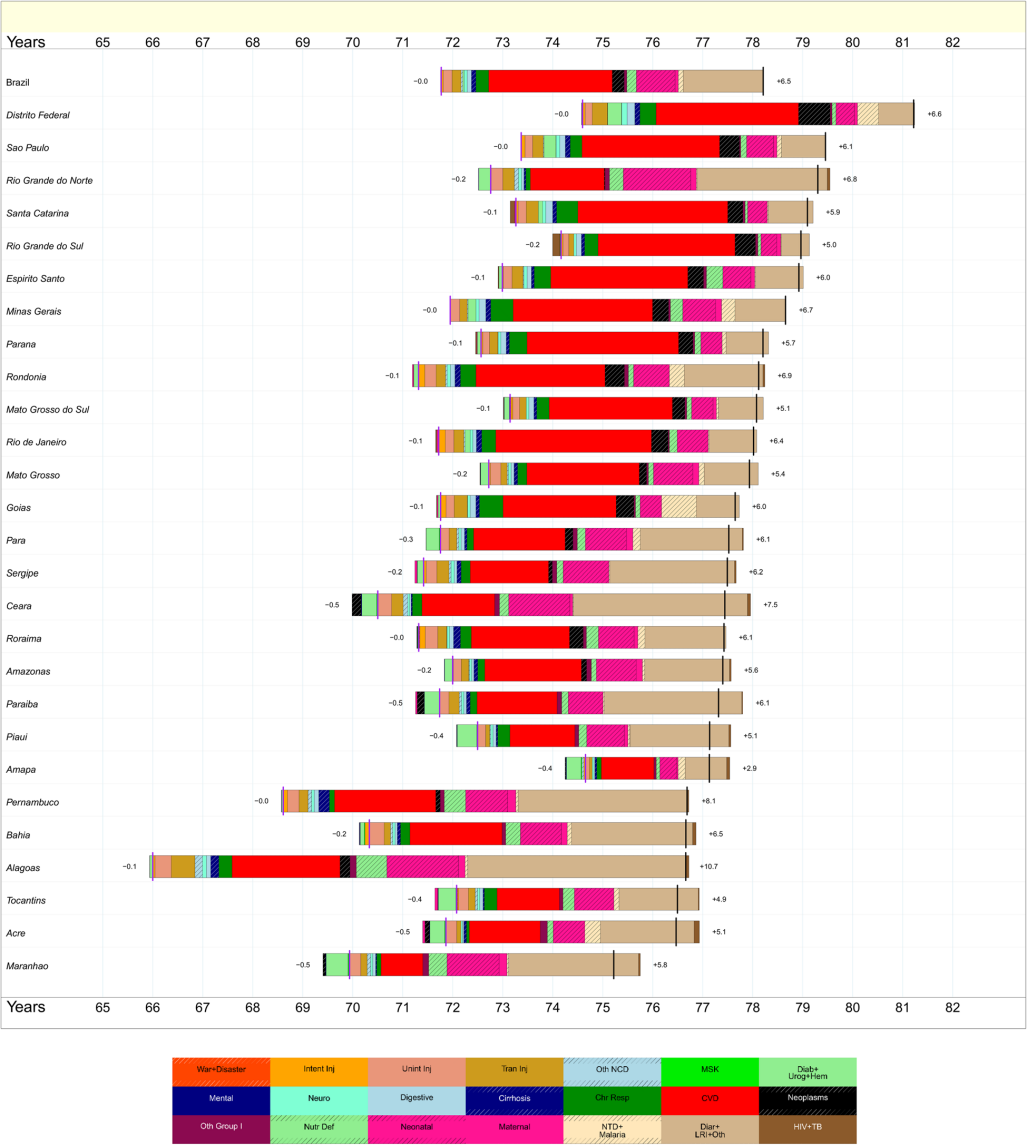
Attribution of changes in life expectancy to changes in major groups of causes of death for females in Brazil’s states, 1990 to 2015. Legend

The decline of mortality rates in Brazil between 1990 and 2015 resulted in an increase of life expectancy from 64.2 to 70.7 years, for males, and from 71.8 to 78.2 for females (Figs. 4 and 5). Life expectancy ranged in 1990 from 58.5 in Maranhão state, for males, to 74.7 in Federal District, for females. In 2015, Maranhão maintained the lowest life expectancy, 67.9 for males, and Federal District presented the highest, 75.2 for females. From 1990 to 2015, a higher increase in life expectancy for men was observed in Maranhão (+9.5 years), Rio de Janeiro (+8.5) and Alagoas (+8.3). The largest increases for females took place in Alagoas (+10.6), Pernambuco (+8.1), and Ceará (+7.0). It is important to highlight the decrease in heterogeneity in life expectancy among states in 2015 compared to 1990.

Some major groups of causes of death were responsible for these changes in life expectancy for Brazil and states. We can attribute to diarrhea, lower respiratory infections, and other infectious diseases (light brown bars on Figs. 4 and 5) the most important contributions for increasing life expectancy in the majority of states from the North and Northeast regions, for males and females. A lower level of cardiovascular diseases in 2015 has contributed to the improvement of life expectancy in all states from the South, Southeast, and Center West regions for males and females. Fewer neonatal disorders have also played an important positive role in changing life expectancy in this period. Increasing numbers of intentional injuries such as violence in men made a negative contribution to life expectancy in 17 states, including many in the Northeast as well as Paraná and Minas Gerais.

### Cause-specific mortality in Brazil and states

A total of 911,317 deaths were estimated in Brazil in 1990, and this number increased to 1,357,434 in 2015. For all causes of death, the total age-standardized mortality rate decreased 28.7% (95% uncertainty interval − 31.1% to −26.1%) over the period from 1102.2 (1085.9–1118.6) to 786.2 per 100,000 (761.2–810.3) for both sexes. At the state level, the decrease varied from 34.9% in Federal District to 6.7% in Amapá (see Additional file 2 for Brazil and Additional files 3, 4, and 5 for states, both sexes, males and females).

Considering the first level of the GBD cause framework, non-communicable diseases (NCDs) accounted for 59.6% of deaths in 1990 and this proportion increased to 75.8% in 2015. On the other hand, communicable, maternal, neonatal, and nutritional disorders (Group 1), which were responsible for 25.6% of all deaths in 1990, declined to cause 11.8% of all deaths in 2015. For injuries, the fraction among all deaths also decreased, from 14.8% in 1990 to 12.4% in 2015. Age-standardized death rates for all three Level 1 groups decreased over the period. The largest decrease occurred for Group 1 disorders, 47.1%. NCDs rates decreased 25.3%, higher than the decline for injury of 22.8%.

**Fig. 6:**
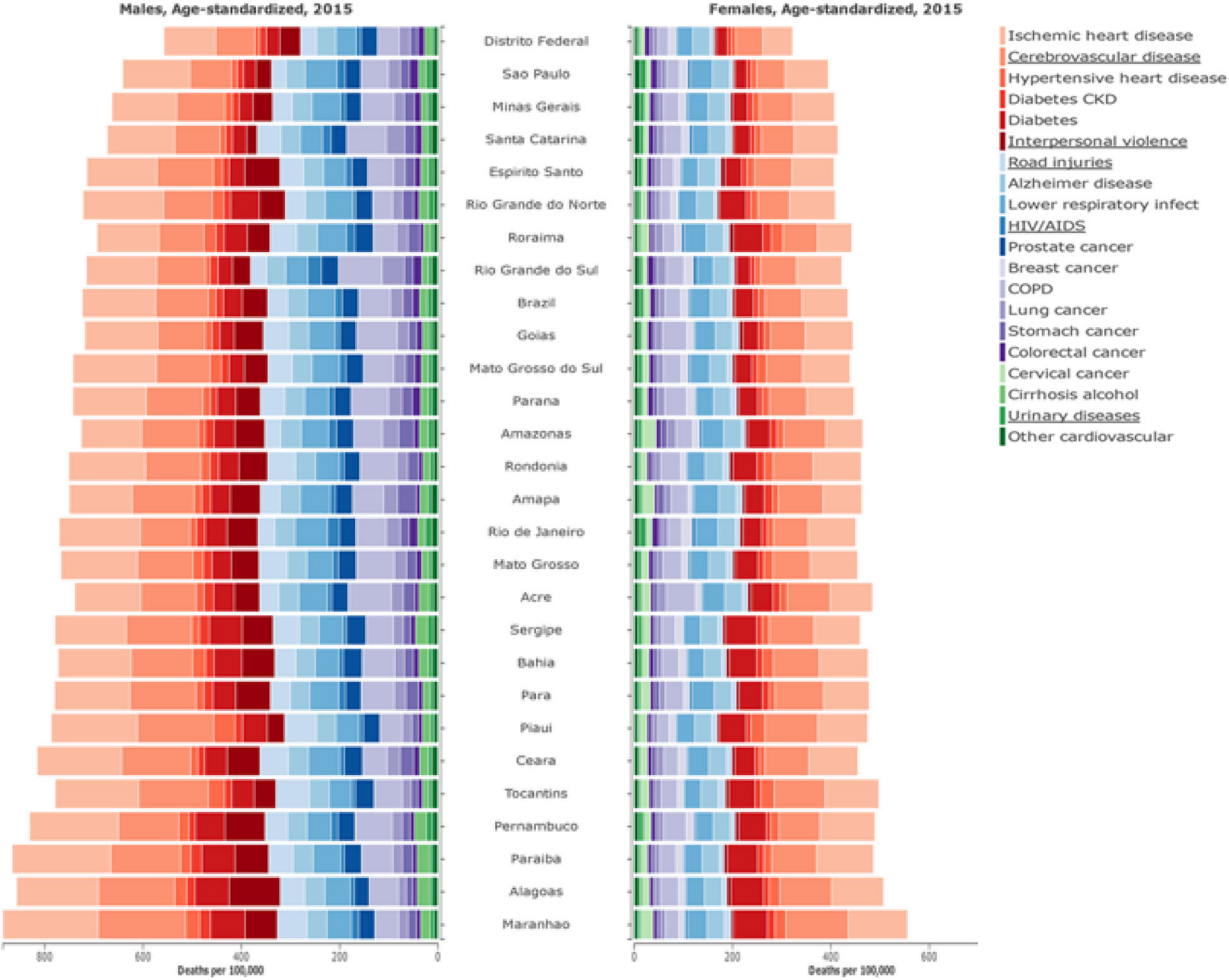
Leading causes of death by sex in Brazil and states, 2015. Legend

**Fig. 7:**
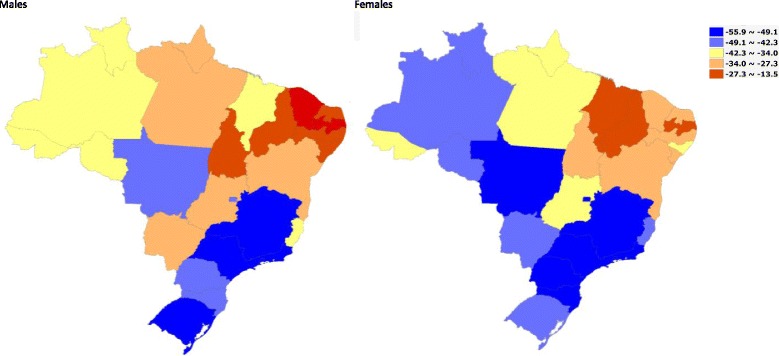
Percent change (%) of age-standardized mortality rates from Ischemic heart disease for males and females in Brazilian states, 1990 and 2015

Figure 6 presents the leading causes of death in Brazil and states in 2015, for males and females. Cardiovascular diseases (CVD) were responsible for 31.2% of all deaths in Brazil in 2015 (see Additional file 2 for Brazil and Additional files 3, 4, and 5 for states, both sexes, males and females). Ischemic heart disease (IHD) is the leading COD in the country and all states, even though presenting a decline of 43.9% in age-standardized rates over the period. Rates for both sexes varied across states from 250.0 in Rio de Janeiro to 161.0 in Sergipe in 1990, and in 2015 from 153.1 per 100,000 in Maranhão to 77.9 in Federal District. During the period, the magnitude of decrease in mortality rates across states and sex varied considerably, from −55.8% for women in Federal District to −7.5% for men in Paraíba (Fig. 7).

Similar to IHD mortality, age-standardized mortality rates due to cerebrovascular disease declined 46.2% considering both sexes. Higher mortality rates of stroke were found in poorer states such as Alagoas and Maranhão when compared to the more developed ones, such as Federal District and São Paulo. On the other hand, hypertensive heart disease had only a slight decrease of age-standardized mortality rates over the period, for both sexes, in the country and for the majority of states.

Cancer in 2015 in Brazil represented 17.4% of total deaths. Age-standardized death rates (per 100,000) were 142.7 and 133.5 in 1990 and 2015, respectively, with a decrease of 6.5% for both sexes combined. Although higher in males than females, the rates also decreased for each sex. Higher rates in 2015 were observed in Paraná, Rio Grande do Sul, Amazonas, Santa Catarina, Amapá, and Ceará states. For males, prostate cancer has become the most important cancer in terms of risk of death in 2015, followed by lung cancer, stomach cancer, and colon/rectum cancer. Breast cancer is the first type of cancer for females, followed by lung cancer, colon/rectum cancer, and cervical cancer. Age-standardized death rates for the principal cancers decreased from 1990 to 2015, except for lung cancer and colon/rectum cancer for females, and for prostate and colon/rectum cancer for males. Higher reductions for those leading cancers were observed in states of the South, Center West, and Southeast regions, particularly in São Paulo (−15.3%) and Federal District (−17.3%). Lesser reductions were observed in general in other states in the North and Northeast regions, and increases were seen in some, such as Paraíba (40.9%, for males; and 13.4% for females), and Ceará (34.6% for males; and 15.2% for females).

Diabetes was among the five top causes of death for females and in the 8th position for males in 2015 (Fig. 6). Diabetes deaths increased by 162.6% over the period 1990 to 2015 for both sexes combined. Although the increase in age-standardized mortality rates was not statistically significant from 1990 to 2015 (4.5%, 95% UI: -1.7-10.3%), a large difference was seen according to sex, with a 3.4% of decrease for women and a 16.2% increase for men. Rates varied markedly across states, from 24.4/100,000 (São Paulo) to 69.5/100,000 (Maranhão) in 2015. States in the Northeast generally had the highest rates, followed by states in the North. The change in age-standardized rates also varied considerably across states over the period, and only five states showed decreasing rates overall - the Federal District, Minas Gerais, Rio de Janeiro, Santa Catarina, and São Paulo.

In addition to these directly attributed deaths, diabetes was also the cause of 59.2% of all chronic kidney disease (CKD) deaths for both sexes combined in 2015. Deaths from CKD due to diabetes increased 208.1% from 1990 to 2015. Age-standardized rates increased 24.4% (15.9–31.7), with the 2015 rate being almost 50% higher in males than in females. Rates across states varied from 10.0/100,000 in the Federal District to 18.3/100,000 in Maranhão. Generally, states in the North had the highest rates in 2015.

Chronic obstructive pulmonary disease (COPD) was among the top five causes of death in 1990 and in 2015. Over the period, COPD mortality rates decreased throughout the country for both sexes combined. Higher rates in 2015 were in Acre, Goiás, Rondônia, Pernambuco and Mato Grosso, close to levels of the Southern states (Paraná, Rio Grande do Sul, and Santa Catarina). Among the top 10 leading COD, Alzheimer disease tripled the number of deaths in 2015, although the risk of death decreased approximately 6% for both sexes combined. Rates for males and females are similar (40.0 and 35.3/100,000, respectively).

Two other non-communicable diseases are among the top 15 causes of death: cirrhosis and other chronic liver diseases due to alcohol use for men, and urinary diseases for women. Deaths due to cirrhosis and other chronic liver diseases due to alcohol use increased 76.6% in Brazil from 1990 to 2015, whereas age-standardized rates fell 17% during the same period. The states of Alagoas, Pernambuco, and Sergipe had the highest age-standardized mortality rates in 2015 (14.7, 13.9, and 13.8 per 100,000, respectively). Urinary diseases were more relevant as COD for the elderly (Additional file 6).

Concerning communicable, maternal, neonatal, and nutritional disorders, LRI and diarrheal diseases were among the top 10 leading causes of death in 1990, but only LRI maintained the rank as a leading cause of death for men and women in 2015, although with decreasing rates for both sexes in Brazil and states. The most important decrease of LRI was for those under-5 (−81.2%), while for the elderly there was a 15.2% increase in age-specific rates for 70 and over (Additional file 7). For diarrheal diseases, the number of deaths and age-standardized mortality rates decreased more than 80% over the period. Changes in age-standardized rates were high in all states in both periods and ranged from −69.3% (Rio Grande do Sul) to −91.1% (Alagoas). In 1990, rates varied markedly across states, from 6.0/100.000 (Rio Grande do Sul) to 86.3/100.000 (Alagoas), with the highest rates in states of the Northeast region followed by states of the North. In 2015, mortality rates decreased across all states, ranging from 1.9/100,000 in Rio Grande do Sul and Rio de Janeiro to 8.0/100,000 in Pernambuco.

As far as the HIV/AIDS and tuberculosis are concerned, an intriguing picture emerges. Percent changes varied according to the specific causes, with a decrease in the number of deaths due to tuberculosis (−27.8%) and an important increase among HIV/AIDS (+260.8%). A similar trend was observed for the age-standardized mortality rates (−64.6% and +112.9% for tuberculosis and HIV/AIDS, respectively).

Deaths from the 3rd GBD group, injuries, were 134,931 in 1990 and 168,018 in 2015, with age-standardized rates declining from 105.1 to 81.2/100,000 in the period. Interpersonal violence (or simply violence) corresponded to 40.1% of cases of injuries in 2015 for men, largely driven by assault with firearms, responsible for the majority of homicides. In 19 of the 27 states there was an increase in death risks due to violence, especially in deaths by firearms. Higher rates in 2015 were found in Alagoas, Pernambuco, Espírito Santo, Pará, Paraíba, Bahia. and Ceará. In the Southeast and Center West regions, Espírito Santo, Mato Grosso, and Goiás were the only states showing an increasing trend.

Mortality rates due to road traffic injuries (RTI) declined during the period, from 36.9/100,000 in 1990 to 24.8/100,000 in 2015 for both sexes, and the decrease was more important among pedestrians (−47.7%) and motor vehicle occupants (−43%). However, pedestrians remain the main victim of RTI in Brazil, with an age-standardized rate of 10.6/100,000 (9.7–11.8) in 2015. Although with lower rates, attention must be brought to the steep increases in mortality risks among motorcyclists (53.3%) and cyclists (33.4%). All states reduced road injury rates between 1990 and 2015, except Piauí. Motorcyclists showed increased rates in all states, with greatest increases in some of the Northeast and Center West states (Piauí, Pernambuco, Ceará, and Mato Grosso do Sul).

### Premature mortality: Years of life lost

**Fig. 8:**
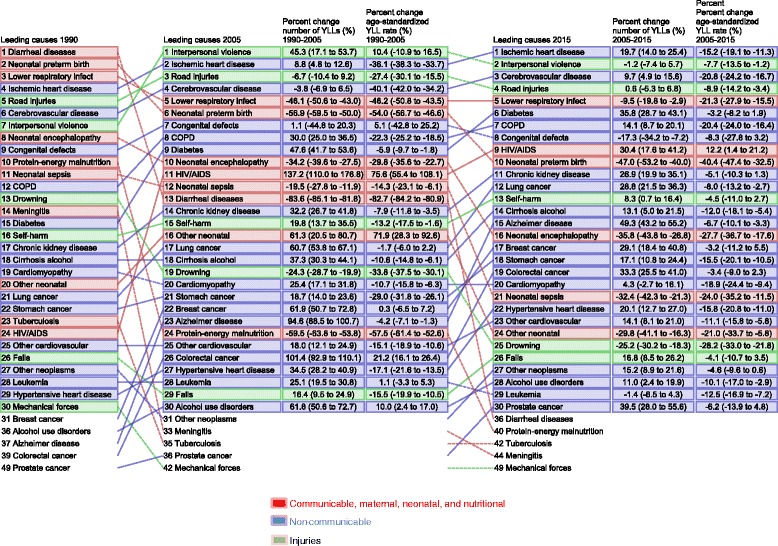
Leading 20 causes of YLLs with median percent change and age-standardized median percent change, all ages, both sexes. Brazil, 1990, 2005, and 2015. Legend

Figure 8 presents the 20 leading causes of premature mortality in Brazil for both sexes and all ages, for two periods, from 1990 to 2005 and from 2005 to 2015. A different epidemiological profile emerges in 2005 and 2015 when compared to 1990. Diarrheal diseases were the leading cause of death in 1990, and dropped to the 13th place in 2005 and 36th in 2015. For the remaining top five leading causes of death from Group 1, neonatal preterm birth moved from the 2th position to 6th then 10th, while LRI changed from 3rd to 5th from 1990 to 2005 but has remained stable since then. On the other hand, violence moved from the 7th position in 1990 to be the first leading cause of premature deaths in 2005, and to the second position in 2015. The maintenance of the five leading causes between 2005 and 2015, despite the change in the order of importance, has confirmed the epidemiologic transition in the country. It is interesting to mention that, although with a small decreasing number of YLLs in the period, the ranking of Alzheimer disease changed from the 16th position in 2000 to 13th in 2015.

We should note the upward shift of HIV/AIDS related deaths in the YLL from 24th place in 1990, to 11th and 9th place in 2005 and 2015, respectively. Among the 20 leading causes of YLL in 2005, HIV/AIDS had the highest median percent increase in both, the number (138%) and the age-standardized YLL (76%), as compared to 1990. On the other hand, YLL due to HIV/AIDS had the 4th largest positive median percent change (behind Alzheimer disease, diabetes, and colorectal cancer) when comparing 2015 to 2005. However, HIV/AIDS was the only cause to show a positive percent change (+12%) in the age-standardized YLL rates in the same comparison.

### Analysis of cause of death by socio-demographic index (SDI)

**Fig. 9:**
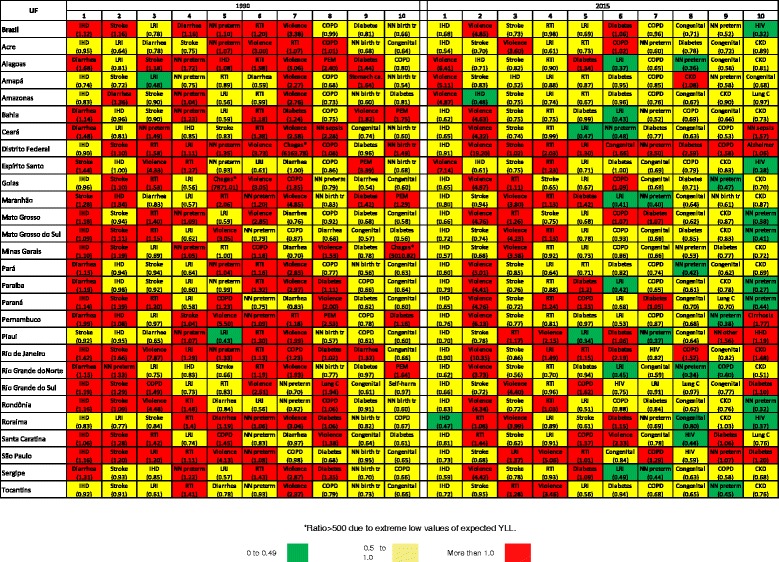
Leading 10 causes of YLLs with the ratio of observed YLLs to expected YLLS given the Socio-demographic Index. Brazil and states, 1990 and 2015

Brazil reduced its total age-standardized YLL rate between 1990 and 2015, bringing its burden of premature deaths closer to expected rates given the country’s SDI. Figure 9 presents ratios of observed YLLs in Brazil and states compared to the average YLLs of countries in the same SDI quintile (expected YLLs) for the leading 10 causes of death in 1990 and 2015, color-coded by the magnitude of ratios between observed and expected YLLs.

In 1990 in Brazil, IHD, stroke, diarrhea, neonatal preterm birth complications, road injury, and violence had YLL ratios higher than expected. In 2015, all causes but violence and diabetes presented YLLs lower than expected on the basis of SDI. Notably, observed premature mortality due to violence was at least twice as high as expected in all states according to the SDI, indicating that this is an important current problem in the whole country. Early deaths due to diabetes also had an observed-to-expected ratio of more than 2.0 in Rio de Janeiro and Federal District. For road injury, the ratio of YLL observed per YLL expected is more than 1.0 in the majority of states in the South, Southeast, and Center West regions, and also in Rondônia, Roraima, Tocantins, Maranhão, and Piauí, from the North and Northeast regions. COPD presented YLLs higher than expected in all states of the South region, in some states from the Center West and Southeast, but in none from the Northeast region.

## Discussion

The results of this study indicate that a widespread reduction on mortality levels occurred in Brazil, with huge progress among children and an important increase in life expectancy. Major shifts in mortality rates have taken place among GBD Level 1 cause groups. In 1990, almost a quarter of all deaths were due to diarrhea, lower respiratory infections, tuberculosis, meningitis, vaccine preventable diseases, and nutritional and neonatal disorders, and were responsible for the majority of premature deaths. In more recent years, the age profile of mortality has shifted to older ages as NCDs are responsible for most deaths in the country. This occurred even though risks of death for the majority of non-communicable diseases decreased over the period.

Brazil has made important progress on a range of the Brazil Millennium Development Goals (MDGs) indicators. The reduction in the proportion of the population living in extreme poverty and in child mortality – with a reduction of almost 50% in the past 15 years, are major achievements from 1990 to 2015 [2, 22]. On the other hand, Brazil is classified at the middle SDI level (SDI = 0.6616) along with other countries such as Bolívia and Indonesia, among others, while within the country only one state was in the first SDI level and five were classified as high-middle SDI. In addition, five states were at the 4th grouping – low-middle SDI level – and the remaining 16 states were at the middle SDI level [11]. Concerning the country’s progress in achieving health-related Sustainable Development Goal (SDG) targets, the nation’s position lags behind several Latin American countries such as Argentina and Chile [23], which poses a challenge for policymakers in Brazil.

The achievements in mortality levels over the last decades could be related to Brazil’s economic and social progress, and to the use of more effective medical technologies and public health interventions [24]. In the health sector, more equitable access to health care has been guaranteed by the expansion of the Family Health Strategy (FHS), with improvements in children’s health, including large and sustained reductions in infant mortality, especially in post-neonatal mortality when due to diarrhea and respiratory infections [5, 25]. Also, FHS development has been associated with reduction of mortality by cardiovascular and cerebrovascular causes [26], and decreasing in hospitalization rates for ambulatory-care–sensitive conditions [27, 28].

The steeper decline of mortality risks due to cardiovascular diseases could be explained by improvements on the risk profile of the population and/or by a better pre-hospital and hospital care for IHD and stroke patients. Indeed, the expansion of the FHS in the period may have improved the control of cardiovascular risk factors, such as high blood pressure and diabetes [5]. Moreover, during the last 25 years, Brazil implemented strong tobacco control policies which reduced its smoking rate by about 50%, with an estimate of 420,000 deaths averted from 1990 to 2010 [29]. A nation-wide ambulance emergency program was launched in 2003 and covered 54% of the population in 2009, which could have improved the quality of pre-hospital care of acute coronary syndrome and stroke patients [30]. Nevertheless, the quality of hospital care continues to be a challenge, and a higher than expected in-hospital mortality has been reported for myocardial infarction and coronary revascularization, especially in the public health system. The decline of age-standardized mortality rates is not uniform and is less impressive in the Northeast and North states, in which there are more non-white inhabitants as well as more poor and vulnerable populations. Previous studies showed that the decline in age-adjusted mortality rates differs according to race, sex, and socioeconomic status with black individuals and lower income populations suffering the greatest impact of CVD [31].

The age-standardized mortality rates for COPD decrease in Brazil similarly to recent worldwide trends of reduction of COPD mortality rates [32], possibly related to highly successful tobacco restriction policies in the country such as increase of taxes and prices on tobacco products, a ban on smoking in public places, health warnings, and marketing restrictions [29]. In 2014, smoking prevalence reduced to 10.8% (95% CI: 10.1–11.4%) in Brazilian capitals [33]. Rates are higher for males and females in Southern states, as found previously [34], probably due to smoking exposure as these states have the highest prevalence of smoking according to the Annual Surveys on Risk and Protective Factors for Chronic Diseases (VIGITEL) results [35]. This finding could be also related to improved data capture as completeness and quality of reporting is better in those states [7]. On the other hand, differential availability of the spirometry test, an important tool for a diagnosis, and the low awareness of COPD by primary health care doctors [36] could contribute to COPD under diagnosis across the country and, consequently, lead to omission of COPD as the real cause of death. Besides, COPD exacerbations could be misclassified as other diseases [37], also possibly resulting in underdiagnosis of COPD and overdiagnosis of LRI. Therefore, the mortality trends in the next decades should be closely monitored since a rise in absolute and age-standardized figures for COPD and LRI could be plausible.

For some other non-communicable diseases such as diabetes and kidney disease however, the results of the GBD 2015 study revealed different shifts in risks. Given that diabetes mortality has declined considerably less than the average across all diseases, diabetes has become increasingly important, a trend that is likely to persist into future years. This finding highlights the importance of providing medical care for the prevention and control of its complications, including CKD. Additionally, since the prevalence of diabetes continues to increase annually [38], sustainable preventive efforts are needed to control disease onset [39]. One community-based intervention designed to prevent chronic diseases was created by the Ministry of Health (*Academia da Cidade* program), aimed at increasing leisure-time physical activity [40], which may contribute to reducing obesity and other diabetes risk factors. Nutrition interventions on dietary risk factors such as diet promotion and obesity prevention have occurred in public policies and in primary health care services over recent years. These include building an integrated inter-ministerial response at the national level to prevent and control obesity, promotion and provision of healthy food in school environments, publishing new national food guidelines and developing the Brazilian Regulation for the Marketing of Food to Infants and Young Children [41].

The relevance of mortality from diseases directly related to alcohol consumption is also a matter of concern. We should point out that aside from cirrhosis and other chronic liver diseases due to alcohol use, other important CODs related to alcohol abuse such as liver cancer due to alcohol disorders and alcohol use disorders further increase the mortality rates. These findings highlight the relevance of alcohol abuse as a COD. Studies show that alcohol-related deaths can be prevented through policies and interventions designed to reduce consumption. However, up to now, Brazil has in general failed to implement policies to control alcohol abuse, as it successfully did with tobacco [42].

Concerning diarrheal diseases and LRI, only the latter remained as a leading cause of death for men and women in 2015. Childhood mortality from LRI was reduced as a result of an increase in wider access to health care, nationwide availability of antibiotics, and vaccination policies. Moreover, the universal availability of influenza vaccines since 1999 and the further introduction of polysaccharide pneumococcal vaccines for children (and in recent years for adults), and a herd protection effect from universal vaccination in children could also have led to a morbidity and mortality reduction in adult pneumococcal pneumonia [43, 44]. The increase of LRI in the elderly could be real or related to misclassification [37], and should further be investigated.

The huge decrease of mortality risks from diarrheal diseases in 2015 was associated with access to improved sanitation [45, 46] and to primary health care^,^ [47]. Access to treated water supply in urban areas and the “Million Cistern Program” in rural areas has reduced the transmission of bacteria and protozoan spread by fecal-oral transmission [48–50]. The introduction of rotavirus vaccination for infants in 2006 contributed to the decline observed in under-5 diarrhea-related mortality and hospitalizations [51].

Also, among the amenable causes of death such as diarrhea, maternal disorders was reduced to one-third of the number of deaths in 1990, and maternal hypertensive disorders was maintained as the leading cause of maternal mortality in 2015. Indirect maternal deaths showed the least improvement in this period and became the second largest contributor to maternal mortality in 2015. Although decreasing, the current high levels of maternal mortality ratios in Brazil, and the huge differences as compared to the developed world, are unacceptable [52].

The epidemiological transition becomes even more evident when we analyze premature deaths. In 1990, the three top leading COD were from communicable, maternal, neonatal, and nutritional disorders (Group 1), while in 2005 and 2015 cardiovascular diseases and injuries stood out. But the maintenance of the top five COD from 2005 to 2015 is an indicator of important challenges to the health system, although the age-standardized YLL rates for these causes have declined significantly during the period. IHD and cerebrovascular diseases, causes of death among the middle aged and the elderly, are quite relevant as interpersonal violence and road traffic injuries, which are causes of death among young people. Although infectious and nutritional diseases related to poverty are declining, we can observe the increasing relative importance of HIV/AIDS; as well as diabetes, chronic kidney disease, lung cancer and Alzheimer disease, the latter related to nutritional and demographic transitions [11]. However, one should bear in mind that any overall appraisal of HIV/AIDS related deaths should be analyzed cautiously. The HIV epidemic is not uniform with regard to region, age, and sex. Furthermore, in countries where the epidemic is mostly concentrated in key populations (e.g., men who have sex with men, injectable drug users, female sex workers) such as Brazil, generalizations may hide the real impact of mortality due to HIV/AIDS in these subgroups. Further analysis of GBD data should be conducted considering the current epidemic scenario, including access to HIV testing, anti-retroviral treatment, and other means of prevention. Finally, the dynamic nature of the epidemic implies careful trend analysis, which may impact public health policies positively or negatively.

Ratios of observed age-standardized YLL rates in comparison with expected YLL based on the Socio-demographic Index (SDI) for all top causes of YLL declined in the country from 1990 to 2015. Violence has persisted as one of the top premature causes of death since 2005, and should be focused as a priority health problem in the country. Despite the decreasing age-standardized YLL homicide rates in 2015, Brazil’s rates still outweigh by far those of the vast majority of countries, although lower than in countries such as South Africa and Colombia [11]. Brazil has a much higher homicides rate due to firearms (19.3 per 100,000 inhabitants in 2015) compared to high-income countries such as France, Canada, and the United States (0.2, 0.5 and 3.6 per 100,000, respectively, in 2010) and even higher than the total homicide epidemic rates estimated for Mexico in recent years. [53, 54].

States in the North, Northeast, and Center West regions had the highest homicide mortality rates. There are multiple factors which contribute to this, such as rapid urbanization and migration of the rural population to urban areas since the 1990s, social disorganization and poverty, associated with drug trafficking, high alcohol consumption, illegal possession of weapons, and, in the North region, serious conflicts over land tenure [55, 56]. Nationwide, there was an increase in homicides rates after the 1980s, and there has been a reduction since 2003, particularly in the São Paulo and Rio de Janeiro states. This decrease may be the result of a combination of socioeconomic measures and public policies that have resulted in improvements in quality of life, higher levels of education, income, and purchasing power of the population [57, 58]. Also, many interventions taken, such as the public policy of disarmament and some poverty reduction programs e.g., the First Job Program and the Bolsa Família Program [59, 60], and the creation of the National Public Security Force in 2004, should be highlighted. These interventions sought to strengthen the state’s presence in regions with high rates of crime, combined with public safety actions, and greater use of intelligence and planning [61].

Another critical problem is RTI, which consumes enormous resources, individually and collectively. Although there was a nationwide decrease, pedestrians remain the main victims of RTI. Brazil has relatively strong national legislation on key aspects of road safety, but a reasonable doubt emerges about its enforcement, especially related to drinking and driving laws [62, 63]. Numbers of motorcyclist road injury deaths increased the most from 1990 to 2015, probably linked to a high rise in the use of this kind of vehicle [64]. This increased motorization is related to the economic growth experienced by developing countries such as Brazil [65] in which increased individual purchasing power was coupled with policies favoring acquisition of private vehicles, especially motorcycles [66].

The GBD study has recognized and discussed extensively the limitations of its approaches [11]. In this study, some specific limitations merit additional attention, particularly those related to the estimation of total number of deaths by age and sex (all-cause mortality). The estimates in the GBD 2015 study indicate that completeness of death counts in Brazil remained around 90% in the 1990s and 2000s, with a slow improvement in the quality of data and, most importantly, in the evolution of mortality rates. Other studies have shown a lower 1990 baseline and a faster increase in completeness levels from 1990 to more recent years, with completeness of total death counts over 95% in the 2000s [19, 67]. These findings highlight the active search of deaths that was systematically implemented in hospitals and non-official sources such as illegal cemeteries in the 2000s with results being used to correct the official mortality statistics [68].

Another issue relates to the redistribution of garbage codes, which is an important acquisition of the GBD approach and largely contributes to correction of issues on data quality, which could affect time trends of specific CODs. For example, the GBD 2015 estimates of higher mortality risks for pedestrians than motorcyclists in 2015 emerged contrary to what might have been expected considering MoH studies [69]. Those estimates could be related to the GBD redistribution algorithms of CODs coded as “unspecified transport accident or victim's mode of transport unknown”, which represented 8.1% of injury deaths and 28.2% of transport injuries in SIM (raw data) in 2014 [70]. The limitations of this study, however, do not affect the major results and the great contribution of the GBD approach to tracking mortality risks in Brazilian states and the challenges to policymakers.

## Conclusions

The GBD 2015 study makes possible the analysis of the epidemiological profile in states with correction for underregistered deaths and redistribution of garbage codes registered as cause of death. The findings indicate that Brazil achieved a widespread reduction on mortality levels from 1990 to 2015, with major progress in deaths among children and an important increase in life expectancy. Major shifts in mortality rates took place among communicable, maternal, neonatal, and nutritional disorders. The mortality age profile has shifted to older ages with increases of NCDs as well as premature deaths due to violence. Thus, policymakers should target priorities for health interventions accordingly.

## Additional files

Additional file 1: Proportions (%) of leading garbage codes registered in the information mortality system SIM by states according to regions. Brazil, 2014. **Table S1** shows the number and proportion (%) of the leading garbage codes in Brazil, and the proportion(%) in each state. (XLSX 25 kb)

Additional file 2: Table A - Number of deaths and age-standardized rates for 249 causes of death, both sexes. Brazil 1990 and 2015. Table B - Number of deaths and age-standardized rates for 249 causes of death, males. Brazil 1990 and 2015. Table C - Number of deaths and age-standardized rates for 249 causes of death, females. Brazil 1990 and 2015. Tables A, B and C present the number of deaths, age-standardized rates and percent change for Brazil, for both sexes, for males and for females, respectively. (DOCX 171 kb)

Additional file 3: Age-standardized mortality rates for 249 causes of death and percent change, both sexes. Brazilian states, 1990 and 2015. Presents the age-standardized rates and percent change for Brazilian states, both sexes, 1990 and 2015. (XLSX 279 kb)

Additional file 4: Age-standardized mortality rates for 249 causes of death and percent change, males. Brazilian states, 1990 and 2015. Presents the age-standardized rates and percent change for Brazilian states, males, 1990 and 2015. (XLSX 272 kb)

Additional file 5: Age-standardized mortality rates for 240 causes of death and percent change, females. Brazilian states, 1990 and 2015. Presents the age-standardized rates and percent change for Brazilian states, females, 1990 and 2015. (XLSX 281 kb)

Additional file 6: Age specific mortality rates (50–69 years and 70 and over) for urinary diseases and male infertility, both sexes, Brazil, 1990 to 2015. mortality rates for age groups 50–69 years and 70 and over for urinary diseases and male infertility, both sexes, Brazil, 1990 to 2015. (PDF 72 kb)

Additional file 7: Trends in under-5 mortality rates due to lower respiratory infections compared to 70 years and over mortality rates in Brazil from 1990 to 2015. Mortality rates (per 100,000) due to lower respiratory infections from 1990 to 2015 among children under 5 years compared to the elderly (70 years and over) in Brazil from 1990 to 2015. (PDF 71 kb)

## Abbreviations

CKD: Chronic kidney disease
COD: Cause of death
CODEm: Cause of Death Ensemble Model
COPD: Chronic obstructive pulmonary disease
CVD: Cardiovascular diseases
DC: Death certificate
DHS: Demographic Health Survey
FHS: Family Health Strategy
GBD: Global Burden of Disease
HDI: Human Development Index
IBGE: Brazilian Institute of Geography and Statistics
ICD-10: International Classification of Diseases – 10th Revision
ICD-9: International Classification of Diseases – 9th Revision
IHD: Ischemic heart disease
LRI: Lower respiratory infections
MoH: Ministry of Health
NCDs: Non-Communicable Diseases
PNAS: National Household Sample Survey
RTI: Road traffic injuries
SDI: Socio-demographic Index
SIM: Mortality Information System [Sistema de Informações sobre Mortalidade]
SUS: Unified Health System [Sistema Único de Saúde]
SVO: Death Investigation Service[Serviço de Verificação de Óbitos]
YLL: Years of life lost
