# Supplementary material for: Cause-specific mortality for 249 causes in Brazil and states during 1990–2015: a systematic analysis for the global burden of disease study 2015
Source: Popul Health Metr. 2017 Nov 22;15:39. doi: 10.1186/s12963-017-0156-y (PMC5700707; doi:10.1186/s12963-017-0156-y)
Supplement: Supplementary file 2 — Table A - Number of deaths and age-standardized rates for 249 causes of death, both sexes. Brazil 1990 and 2015. Table B - Number of deaths and age-standardized rates for 249 causes of death, males. Brazil 1990 and 2015. Table C - Number of deaths and age-standardized rates for 249 causes of death, females. Brazil 1990 and 2015. Tables A, B and C present the number of deaths, age-standardized rates and percent change for Brazil, for both sexes, for males and for females, respectively. (DOCX 171 kb) [file 12963_2017_156_MOESM2_ESM.docx]

**Additional file 2: Table A - Number of deaths and age-standardized rates for 249 causes of death, both sexes. Brazil 1990 and 2015.**

| **Causes of death** | **Number of deaths** | | | **Age-standardized death rates (per 100,000)** | | |
| --- | --- | --- | --- | --- | --- | --- |
|  | **1990** | **2015** | **% change 1990-2015** | **1990** | **2015** | **% change 1990-2015** |
| **All causes** | 911,317  (896,944-925,847) | 1,357,434  (1,312,612-1,400,906) | 49  (43.8-54.5) | 1102.2  (1085.9-1118.6) | 786.2  (761.2-810.3) | -28.7  (-31.1--26.1) |
| **A - Communicable, maternal, neonatal, and nutritional diseases** | 233,554  (222,998-240,244) | 159,917  (139,858-170,945) | -31.1  (-38.7--26.5) | 178.5  (168.3-183.4) | 93.8  (81.4-100.7) | -47.1  (-52.7--43.6) |
| A.1 - HIV/AIDS and tuberculosis | 13,740  (11,664-15,402) | 26,812  (23,412-31,197) | 94.9  (62.5-140.2) | 12.8  (10.7-14.8) | 12.5  (10.9-14.6) | -2.6  (-19.2-20.3) |
| A.1.1 - Tuberculosis | 7,904  (5,756-9,483) | 5,763  (3,947-7,379) | -27.8  (-46--2.8) | 8.3  (6.2-10.3) | 3  (2-3.8) | -64.6  (-74.3--52) |
| A.1.2 - HIV/AIDS | 5,836  (5,464-6,337) | 21,050  (17,894-25,146) | 260.8  (184.5-348.6) | 4.5  (4.2-4.8) | 9.5  (8.1-11.5) | 112.9  (69.1-168.8) |
| A.1.2.1 - HIV/AIDS - Tuberculosis | 1,525  (1,046-1,796) | 1,961  (1,282-2,360) | 27.1  (-6.1-71.8) | 1.2  (0.8-1.4) | 0.9  (0.6-1.1) | -24.9  (-44.6-1.7) |
| A.1.2.2 - HIV/AIDS resulting in other diseases | 4,311  (3,837-4,934) | 19,089  (15,888-23,261) | 344.3  (234.9-480.8) | 3.3  (2.9-3.8) | 8.7  (7.2-10.6) | 162.7  (98.9-245.4) |
| **A.2 - Diarrhea, lower respiratory, and other common infectious diseases** | 119,418  (111,562-124,075) | 85,657  (65,814-94,532) | -27.3  (-41.7--20.4) | 101.6  (92.4-105.8) | 52.9  (40.7-58.6) | -47.2  (-56.6--42.1) |
| A.2.1 - Diarrheal diseases | 46,035  (43,480-48,424) | 6,343  (5,942-6,732) | -86.2  (-87.3--85) | 29.6  (28.3-31) | 3.9  (3.6-4.2) | -86.8  (-87.8--85.7) |
| A.2.2 - Intestinal infectious diseases | 139  (105-197) | 55  (32-92) | -60.1  (-76.3--48.3) | 0.1  (0.1-0.1) | 0  (0-0) | -70.4  (-81.7--63.6) |
| A.2.2.1 - Typhoid fever | 55  (26-100) | 41  (20-76) | -24.9  (-29.6--19.2) | 0  (0-0.1) | 0  (0-0) | -53.7  (-56.3--50.9) |
| A.2.2.2 - Paratyphoid fever | 0  (0-1) | 2  (1-3) | 277.9  (197.5-363.8) | 0  (0-0) | 0  (0-0) | 145.1  (92.1-209.1) |
| A.2.2.3 - Other intestinal infectious diseases | 84  (69-127) | 12  (2-20) | -85.7  (-97.8--74.1) | 0.1  (0-0.1) | 0  (0-0) | -87.2  (-98.1--77.1) |
| A.2.3 - Lower respiratory infections | 59,599  (53,934-62,258) | 75,602  (55,633-84,416) | 29.6  (1.6-42.1) | 63.5  (55.9-67.3) | 47  (34.7-52.7) | -24.4  (-39--17) |
| A.2.4 - Upper respiratory infections | 48  (38-61) | 45  (37-56) | -5.5  (-31.4-29.4) | 0  (0-0.1) | 0  (0-0) | -47.2  (-62.7--25.1) |
| A.2.5 - Otitis media | 205  (187-228) | 50  (45-56) | -75.8  (-79--71.6) | 0.1  (0.1-0.1) | 0  (0-0) | -78.3  (-81.2--74.4) |
| A.2.6 - Meningitis | 8,027  (6,256-8,844) | 2,785  (2,515-3,530) | -67.2  (-70.4--49.9) | 4.8  (3.7-5.2) | 1.5  (1.3-1.9) | -70.7  (-73.4--55.8) |
| A.2.6.1 - Pneumococcal meningitis | 1,460  (1,048-1,856) | 432  (356-571) | -71.8  (-76--56.6) | 0.8  (0.6-1.1) | 0.2  (0.2-0.3) | -73.8  (-77.4--60.1) |
| A.2.6.2 - H influenzae type B meningitis | 1,018  (695-1,363) | 97  (75-134) | -90.9  (-92.7--84.6) | 0.6  (0.4-0.7) | 0.1  (0-0.1) | -90.6  (-92.3--84.2) |
| A.2.6.3 - Meningococcal meningitis | 2,648  (1,979-3,245) | 982  (838-1,271) | -64.7  (-69.3--46) | 1.6  (1.2-1.9) | 0.5  (0.4-0.7) | -67.8  (-71.4--51.7) |
| A.2.6.4 - Other meningitis | 2,900  (2,200-3,368) | 1,274  (1,127-1,605) | -58.2  (-63--37.2) | 1.8  (1.4-2.1) | 0.7  (0.6-0.9) | -65.2  (-68.7--48.2) |
| A.2.7 - Encephalitis | 840  (690-901) | 445  (415-506) | -48.2  (-52.5--32.3) | 0.6  (0.5-0.6) | 0.2  (0.2-0.3) | -58.4  (-61.6--44.7) |
| A.2.8 - Diphtheria | 32  (20-51) | 4  (3-5) | -87.1  (-92.4--78.4) | 0  (0-0) | 0  (0-0) | -88.1  (-92.9--80.2) |
| A.2.9 - Whooping cough | 1,938  (856-3,726) | 86  (22-266) | -96.1  (-98.9--84.9) | 1  (0.4-1.9) | 0.1  (0-0.2) | -95.4  (-98.7--82.2) |
| A.2.10 - Tetanus | 1,137  (790-1,254) | 192  (123-628) | -87.3  (-89.7--46.4) | 0.9  (0.6-1) | 0.1  (0.1-0.3) | -91.9  (-93.4--65.1) |
| A.2.11 - Measles | 1,109  (806-1,492) | 3  (2-5) | -99.7  (-99.8--99.6) | 0.6  (0.4-0.7) | 0  (0-0) | -99.7  (-99.7--99.5) |
| A.2.12 - Varicella and herpes zoster | 307  (242-390) | 45  (33-64) | -85.5  (-90--77.3) | 0.3  (0.2-0.5) | 0  (0-0) | -91.8  (-95.1--86.4) |
| **A.3 - Neglected tropical diseases and malaria** | 9,541 (9,121-9,962) | 7,486 (6,918-8,049) | -21.4 (-27.9--14.9) | 10.9 (10.4-11.4) | 4.2 (3.9-4.5) | -61.7 (-65--58.4) |
| A.3.1 - Malaria | 771  (657-909) | 76  (58-100) | -90.3  (-92.8--86.2) | 0.5  (0.4-0.6) | 0  (0-0) | -92.3  (-94.3--89.1) |
| A.3.2 - Chagas disease | 7,065  (6,752-7,400) | 5,952  (5,498-6,453) | -15.7  (-23.4--7.7) | 8.7  (8.3-9.1) | 3.4  (3.1-3.7) | -61.3  (-64.7--57.6) |
| A.3.3 - Leishmaniasis | 327  (264-419) | 471  (380-597) | 43.9  (31.6-59.4) | 0.3  (0.2-0.3) | 0.2  (0.2-0.3) | -8.4  (-15.9-0.8) |
| A.3.3.1 - Visceral leishmaniasis | 327  (264-419) | 471  (380-597) | 43.9  (31.6-59.4) | 0.3  (0.2-0.3) | 0.2  (0.2-0.3) | -8.4  (-15.9-0.8) |
| A.3.4 - African trypanosomiasis | NA  (NA-NA) | NA  (NA-NA) | NA  (NA-NA) | NA  (NA-NA) | NA  (NA-NA) | NA  (NA-NA) |
| A.3.5 - Schistosomiasis | 923  (777-1,084) | 271  (225-328) | -70.7  (-77.4--61.8) | 1.1  (0.9-1.3) | 0.1  (0.1-0.2) | -86.9  (-90--82.7) |
| A.3.6 - Cysticercosis | 157  (151-162) | 37  (34-41) | -76.1  (-78.2--74.1) | 0.1  (0.1-0.1) | 0  (0-0) | -87.3  (-88.4--86.2) |
| A.3.7 - Cystic echinococcosis | 16  (15-17) | 6  (5-6) | -63.2  (-65.5--60.3) | 0  (0-0) | 0  (0-0) | -80.5  (-81.8--79) |
| A.3.11 - Dengue | 30  (25-51) | 505  (249-689) | 1738  (458.4-2449.6) | 0  (0-0) | 0.3  (0.1-0.4) | 1092.3  (248.8-1541.5) |
| A.3.12 - Yellow fever | 17  (4-44) | 6  (1-17) | -62.2  (-65.9--57.9) | 0  (0-0) | 0  (0-0) | -71.5  (-74--68.4) |
| A.3.13 - Rabies | 59  (42-66) | 4  (3-5) | -93.6  (-94.8--85.3) | 0  (0-0) | 0  (0-0) | -95.1  (-95.9--88.5) |
| A.3.14 - Intestinal nematode infections | 34  (22-49) | 16  (8-28) | -54.5  (-78.5--5) | 0  (0-0) | 0  (0-0) | -57.1  (-80.7--11.1) |
| A.3.14.1 - Ascariasis | 34  (22-49) | 16  (8-28) | -54.5  (-78.5--5) | 0  (0-0) | 0  (0-0) | -57.1  (-80.7--11.1) |
| A.3.17 - Ebola | NA  (NA-NA) | NA  (NA-NA) | NA  (NA-NA) | NA  (NA-NA) | NA  (NA-NA) | NA  (NA-NA) |
| A.3.19 - Other neglected tropical diseases | 143  (111-182) | 140  (79-170) | 2.1  (-43.4-21.7) | 0.1  (0.1-0.1) | 0.1  (0-0.1) | -13.3  (-50.6-3.2) |
| **A.4 - Maternal disorders** | 3,081  (2,896-3,299) | 1,972  (1,786-2,165) | -36  (-43.2--27.9) | 2  (1.8-2.1) | 0.9  (0.8-0.9) | -56  (-61--50.4) |
| A.4.1 - Maternal hemorrhage | 520  (454-585) | 289  (239-348) | -44.6  (-55.4--30.2) | 0.3  (0.3-0.4) | 0.1  (0.1-0.2) | -62.6  (-69.9--53.2) |
| A.4.2 - Maternal sepsis and other maternal infections | 443  (386-508) | 237  (192-287) | -46.7  (-58.4--31.2) | 0.3  (0.2-0.3) | 0.1  (0.1-0.1) | -63.6  (-71.5--52.9) |
| A.4.3 - Maternal hypertensive disorders | 737  (667-822) | 403  (337-480) | -45.6  (-55.9--33.5) | 0.5  (0.4-0.5) | 0.2  (0.1-0.2) | -61.2  (-68.4--52.8) |
| A.4.4 - Maternal obstructed labor and uterine rupture | 55  (44-68) | 37  (26-49) | -33.3  (-54.1--5.1) | 0  (0-0) | 0  (0-0) | -56.3  (-69.8--37.8) |
| A.4.5 - Maternal abortion, miscarriage, and ectopic pregnancy | 458  (390-520) | 250  (209-300) | -45.5  (-52.9--37.2) | 0.3  (0.3-0.4) | 0.1  (0.1-0.1) | -64.7  (-69.9--59.4) |
| A.4.6 - Indirect maternal deaths | 439  (375-517) | 403  (342-472) | -8.1  (-28.2-16.6) | 0.3  (0.2-0.3) | 0.2  (0.1-0.2) | -34.6  (-49--17.8) |
| A.4.7 - Late maternal deaths | 100  (77-128) | 89  (70-112) | -10.4  (-33.8-21.2) | 0.1  (0-0.1) | 0  (0-0) | -38.1  (-53.8--16.7) |
| A.4.8 - Maternal deaths aggravated by HIV/AIDS | 1  (1-2) | 2  (1-3) | 56.2  (18.8-92.8) | 0  (0-0) | 0  (0-0) | 7.9  (-17.6-33.4) |
| A.4.9 - Other maternal disorders | 326  (294-361) | 262  (221-308) | -20.1  (-33.9--3.8) | 0.2  (0.2-0.2) | 0.1  (0.1-0.1) | -44.4  (-54--33.3) |
| **A.5 - Neonatal disorders** | 69,532  (67,501-71,533) | 25,137  (22,843-27,716) | -63.9  (-67.3--59.8) | 35.8  (34.7-36.8) | 15.5  (14.1-17.1) | -56.7  (-60.7--51.8) |
| A.5.1 - Neonatal preterm birth complications | 41,385 (37,696-43,812) | 9,588 (8,294-11,230) | -77 (-79.8--72.1) | 21.3 (19.4-22.5) | 5.9 (5.1-6.9) | -72.4 (-75.8--66.5) |
| A.5.2 - Neonatal encephalopathy due to birth asphyxia and trauma | 13,784  (12,711-15,145) | 5,834  (5,029-6,818) | -57.7  (-63.2--51.1) | 7.1  (6.5-7.8) | 3.6  (3.1-4.2) | -49.3  (-55.9--41.4) |
| A.5.3 - Neonatal sepsis and other neonatal infections | 9,421  (6,283-13,032) | 5,112  (3,594-7,114) | -45.5  (-53.5--36.9) | 4.9  (3.2-6.7) | 3.2  (2.2-4.4) | -34.8  (-44.3--24.5) |
| A.5.4 - Hemolytic disease and other neonatal jaundice | 1,026  (914-1,203) | 199  (159-242) | -80.7  (-84.6--75.9) | 0.5  (0.5-0.6) | 0.1  (0.1-0.1) | -76.8  (-81.6--71.1) |
| A.5.5 - Other neonatal disorders | 3,916  (3,511-4,619) | 4,405  (3,409-5,215) | 14.2  (-20.8-38.6) | 2  (1.8-2.4) | 2.7  (2.1-3.2) | 36.8  (-5.1-66.2) |
| **A.6 - Nutritional deficiencies** | 13,570  (10,445-14,964) | 9,568  (7,584-10,585) | -29.4  (-35.5--23.1) | 12.2  (9.7-13.4) | 6  (4.8-6.7) | -50.2  (-54.1--46.3) |
| A.6.1 - Protein-energy malnutrition | 12,738  (9,782-14,080) | 8,822  (6,968-9,850) | -30.6  (-36.8--23.9) | 11.4  (9.2-12.5) | 5.6  (4.4-6.2) | -50.7  (-54.7--46.7) |
| A.6.2 - Iodine deficiency | 27  (16-50) | 20  (12-35) | -25.7  (-66.2-58.5) | 0  (0-0) | 0  (0-0) | -54.8  (-79-2.8) |
| A.6.4 - Iron-deficiency anemia | 17  (10-34) | 29  (12-41) | 89.7  (-6.2-116.5) | 0  (0-0) | 0  (0-0) | 45.7  (-36.1-66) |
| A.6.5 - Other nutritional deficiencies | 787  (595-961) | 696  (495-863) | -11.9  (-24.8-5.8) | 0.8  (0.6-1) | 0.4  (0.3-0.5) | -44.7  (-53.5--34.8) |
| **A.7 - Other communicable, maternal, neonatal, and nutritional diseases** | 4,672  (3,734-5,905) | 3,285  (2,986-3,510) | -29.2  (-43.2--13.1) | 3.3  (2.8-4) | 1.7  (1.6-1.8) | -48.2  (-55.9--40.3) |
| A.7.1 - Sexually transmitted diseases excluding HIV | 2,293  (1,370-3,505) | 454  (333-602) | -79.9  (-84.8--72.5) | 1.3  (0.8-1.9) | 0.3  (0.2-0.3) | -79.6  (-84.1--73.4) |
| A.7.1.1 - Syphilis | 2,267  (1,343-3,480) | 424  (307-571) | -81.1  (-85.5--74.3) | 1.2  (0.8-1.8) | 0.2  (0.2-0.3) | -80.5  (-84.6--74.7) |
| A.7.1.2 - Chlamydial infection | 4  (4-5) | 5  (3-6) | 32.6  (-26.2-52.4) | 0  (0-0) | 0  (0-0) | -29.7  (-62.5--18.4) |
| A.7.1.3 - Gonococcal infection | 14  (13-18) | 17  (11-20) | 27.2  (-29.7-45.2) | 0  (0-0) | 0  (0-0) | -35.7  (-65.7--26.1) |
| A.7.1.6 - Other sexually transmitted diseases | 8  (7-10) | 8  (5-10) | 12.2  (-36.2-28.1) | 0  (0-0) | 0  (0-0) | -39.8  (-66.7--31) |
| A.7.2 - Hepatitis | 1,108  (1,064-1,152) | 1,236  (1,150-1,325) | 11.4  (2.8-20.8) | 1  (0.9-1) | 0.6  (0.6-0.7) | -35.8  (-40.6--30.4) |
| A.7.2.1 - Acute hepatitis A | 282  (185-373) | 106  (43-193) | -64  (-77.1--46.4) | 0.1  (0.1-0.2) | 0.1  (0-0.1) | -65.3  (-76.3--50.2) |
| A.7.2.2 - Acute hepatitis B | NA | NA | NA  (NA-NA) | NA | NA | NA  (NA-NA) |
| A.7.2.3 - Acute hepatitis C | NA | NA | NA  (NA-NA) | NA | NA | NA  (NA-NA) |
| A.7.2.4 - Acute hepatitis E | NA  (NA-NA) | NA  (NA-NA) | NA  (NA-NA) | NA  (NA-NA) | NA  (NA-NA) | NA  (NA-NA) |
| A.7.4 - Other infectious diseases | 1,270  (1,115-1,395) | 1,595  (1,342-1,732) | 26.3  (10.4-37.9) | 1.1  (1-1.2) | 0.8  (0.7-0.9) | -23.1  (-32.8--15.8) |
| **B - Non-communicable diseases** | 542,832  (532,149-554,906) | 1,029,499  (992,362-1,068,244) | 89.7  (82.4-97.2) | 818.6  (803.9-834.7) | 611.3  (589.8-633.9) | -25.3  (-28--22.6) |
| **B.1 - Neoplasms** | 105,275  (102,761-109,195) | 236,345  (226,265-248,290) | 124.5  (115.4-134.9) | 142.7  (139.2-149.1) | 133.5  (127.9-140.4) | -6.5  (-10.1--2.3) |
| B.1.1 - Lip and oral cavity cancer | 2,102  (2,015-2,190) | 4,915  (4,644-5,201) | 134.1  (118.2-150.4) | 2.8  (2.7-2.9) | 2.7  (2.5-2.8) | -5.2  (-11.3-1.3) |
| B.1.2 - Nasopharynx cancer | 217  (203-246) | 528  (406-570) | 147.4  (91.4-165.9) | 0.2  (0.2-0.3) | 0.3  (0.2-0.3) | 18.7  (-8.3-27.7) |
| B.1.3 - Other pharynx cancer | 1,473  (1,403-1,546) | 3,356  (3,099-3,617) | 127.5  (109.2-148.4) | 1.9  (1.8-1.9) | 1.8  (1.6-1.9) | -5  (-12.6-3.4) |
| B.1.4 - Esophageal cancer | 5,278 (5,073-5,475) | 10,896 (10,253-11,609) | 106.2 (93-123) | 7.2 (7-7.5) | 6 (5.6-6.4) | -17.2 (-22.4--10.8) |
| B.1.5 - Stomach cancer | 13,554  (13,159-13,943) | 21,466  (20,434-22,600) | 58.3  (49.5-67.2) | 19.8  (19.3-20.4) | 12.3  (11.7-13) | -37.9  (-41.2--34.3) |
| B.1.6 - Colon and rectum cancer | 6,894  (6,688-7,121) | 21,419  (20,140-22,725) | 210.5  (192.6-229.9) | 10.3  (10-10.6) | 12.4  (11.6-13.2) | 20.1  (13-27.5) |
| B.1.7 - Liver cancer | 4,560  (4,266-5,183) | 10,393  (9,616-12,058) | 127.6  (109.3-149) | 6.4  (5.9-7.3) | 5.9  (5.4-6.8) | -8.8  (-15.9-0.2) |
| B.1.7.1 - Liver cancer due to hepatitis B | 1,086  (999-1,246) | 2,058  (1,867-2,381) | 89.4  (75.3-104.6) | 1.3  (1.2-1.5) | 1.1  (1-1.2) | -16.4  (-22.3--9.4) |
| B.1.7.2 - Liver cancer due to hepatitis C | 1,637  (1,511-1,870) | 3,609  (3,301-4,288) | 119.8  (100.5-145.1) | 2.5  (2.3-2.9) | 2.1  (1.9-2.5) | -16.4  (-23.4--6.1) |
| B.1.7.3 - Liver cancer due to alcohol use | 1,089  (997-1,234) | 3,326  (3,040-3,755) | 205.6  (183.3-229.5) | 1.5  (1.4-1.7) | 1.9  (1.7-2.1) | 21.2  (12.4-30.5) |
| B.1.7.4 - Liver cancer due to other causes | 749  (693-865) | 1,400  (1,271-1,668) | 86  (68.6-108.1) | 1.1  (1-1.2) | 0.8  (0.7-1) | -25.5  (-32.1--16.3) |
| B.1.8 - Gallbladder and biliary tract cancer | 2,942  (2,831-3,049) | 4,871  (4,377-5,369) | 66  (47.9-82.7) | 4.4  (4.3-4.6) | 2.8  (2.5-3.1) | -36.1  (-43.1--29.5) |
| B.1.9 - Pancreatic cancer | 4,125  (4,009-4,253) | 11,344  (10,767-12,019) | 174.7  (159.6-191.8) | 6.1  (5.9-6.3) | 6.6  (6.2-6.9) | 7  (1-13.9) |
| B.1.10 - Larynx cancer | 2,346  (2,241-2,453) | 4,773  (4,467-5,119) | 103.5  (88.2-120.2) | 3.1  (2.9-3.2) | 2.6  (2.4-2.8) | -16  (-22.2--9.5) |
| B.1.11 - Tracheal, bronchus, and lung cancer | 13,495  (13,082-13,905) | 32,296  (30,514-34,200) | 139.2  (125.7-155.1) | 18.7  (18.1-19.3) | 18.3  (17.3-19.4) | -2.3  (-7.6-4.3) |
| B.1.12 - Malignant skin melanoma | 728  (560-787) | 1,994  (1,529-2,183) | 173.7  (155.1-193.3) | 0.9  (0.7-1) | 1.1  (0.8-1.2) | 20.4  (11.7-29.4) |
| B.1.13 - Non-melanoma skin cancer | 864  (829-901) | 2,705  (2,536-2,888) | 213.1  (190.8-238) | 1.3  (1.3-1.4) | 1.6  (1.5-1.8) | 22.7  (13.6-33.4) |
| B.1.13.1 - Non-melanoma skin cancer (squamous-cell carcinoma) | 864  (829-901) | 2,705  (2,536-2,888) | 213.1  (190.8-238) | 1.3  (1.3-1.4) | 1.6  (1.5-1.8) | 22.7  (13.6-33.4) |
| B.1.14 - Mesothelioma | 759  (532-865) | 672  (620-758) | -10.6  (-27.2-44.1) | 1.1  (0.7-1.3) | 0.4  (0.3-0.4) | -67.9  (-74.9--42.3) |
| B.1.15 - Breast cancer | 7,340  (6,261-7,770) | 17,304  (15,205-18,728) | 136.2  (112-159.9) | 9.1  (7.7-9.6) | 9.2  (8-9.9) | 1  (-9.6-11.6) |
| B.1.16 - Cervical cancer | 6,592  (6,318-6,883) | 10,027  (9,379-10,740) | 52.3  (40.5-64.2) | 7.9  (7.6-8.3) | 5.3  (4.9-5.6) | -33.8  (-38.9--28.4) |
| B.1.17 - Uterine cancer | 1,200  (1,140-1,265) | 2,321  (2,152-2,526) | 93.1  (76.1-112.2) | 1.8  (1.7-1.9) | 1.3  (1.2-1.5) | -24.7  (-31.3--17.1) |
| B.1.18 - Ovarian cancer | 1,868  (1,780-1,962) | 4,455  (4,156-4,764) | 138.6  (119.8-158.1) | 2.3  (2.2-2.4) | 2.4  (2.2-2.6) | 3.3  (-5-11.7) |
| B.1.19 - Prostate cancer | 6,454  (5,305-9,337) | 21,130  (17,225-27,721) | 233.7  (185.4-263) | 11.8  (9.8-17.1) | 13.4  (10.9-17.7) | 15.5  (-0.5-26.2) |
| B.1.20 - Testicular cancer | 189  (176-203) | 337  (307-371) | 77.8  (59.9-97) | 0.2  (0.1-0.2) | 0.2  (0.1-0.2) | 2.3  (-7.7-12.7) |
| B.1.21 - Kidney cancer | 1,253  (1,210-1,300) | 3,736  (3,493-3,975) | 198.5  (177.6-218.6) | 1.5  (1.5-1.6) | 2.1  (2-2.2) | 35.5  (25.7-44.9) |
| B.1.22 - Bladder cancer | 1,821  (1,761-1,892) | 4,795  (4,530-5,088) | 162.8  (146.5-181.9) | 3  (2.9-3.2) | 2.9  (2.8-3.1) | -3.2  (-9-4.4) |
| B.1.23 - Brain and nervous system cancer | 2,749  (2,504-3,870) | 8,960  (6,398-9,958) | 256.5  (77.3-291.5) | 2.7  (2.5-3.8) | 4.7  (3.3-5.2) | 90.3  (-7.6-108.3) |
| B.1.24 - Thyroid cancer | 514  (411-549) | 968  (877-1,134) | 86.1  (72.5-130) | 0.7  (0.6-0.8) | 0.6  (0.5-0.7) | -26.3  (-31.7--9.3) |
| B.1.25 - Hodgkin lymphoma | 655  (460-726) | 743  (592-940) | 11  (1.6-34.5) | 0.7  (0.5-0.7) | 0.4  (0.3-0.5) | -43.1  (-47.8--30.9) |
| B.1.26 - Non-Hodgkin lymphoma | 2,644 (2,281-2,981) | 6,035 (4,600-6,660) | 131.6 (76.3-149.3) | 3 (2.6-3.5) | 3.3 (2.5-3.7) | 12.5 (-16.5-21.7) |
| B.1.27 - Multiple myeloma | 959  (926-996) | 3,246  (3,069-3,440) | 238.7  (216.4-260.5) | 1.3  (1.2-1.3) | 1.8  (1.7-1.9) | 40.9  (32.1-50.4) |
| B.1.28 - Leukemia | 5,319  (5,142-5,489) | 9,293  (8,845-9,731) | 74.7  (65.6-84) | 5.2  (5-5.3) | 5.1  (4.8-5.4) | -1.4  (-6.7-3.9) |
| B.1.28.1.1 - Acute lymphoid leukemia | 1,778  (1,609-1,972) | 2,205  (1,907-2,555) | 24  (13.4-35.9) | 1.2  (1.1-1.4) | 1.1  (1-1.3) | -8.6  (-16.1-0.3) |
| B.1.28.1.2 - Chronic lymphoid leukemia | 430  (395-478) | 1,304  (1,142-1,479) | 203.4  (173.8-234.7) | 0.7  (0.7-0.8) | 0.8  (0.7-0.9) | 13.9  (2.1-26.2) |
| B.1.28.2.1 - Acute myeloid leukemia | 2,267  (1,992-2,465) | 4,722  (4,130-5,133) | 108.4  (93.9-122.7) | 2.2  (2-2.4) | 2.5  (2.2-2.8) | 14.5  (6.2-22.9) |
| B.1.28.2.2 - Chronic myeloid leukemia | 844  (770-930) | 1,062  (922-1,210) | 25.7  (13-38.2) | 1  (0.9-1.1) | 0.6  (0.5-0.7) | -39.1  (-45.5--32.7) |
| B.1.29 - Other neoplasms | 6,377  (5,711-6,755) | 11,367  (10,583-13,239) | 76.1  (64.7-102.7) | 7  (6.3-7.5) | 6.2  (5.8-7.2) | -12.3  (-17.8--0.8) |
| **B.2 - Cardiovascular diseases** | 267,634  (262,150-273,226) | 424,058  (407,250-444,686) | 58.2  (51.7-66.3) | 429.5  (421.1-438.1) | 256  (246.3-268.3) | -40.5  (-42.9--37.5) |
| B.2.1 - Rheumatic heart disease | 2,608  (2,520-2,705) | 2,744  (2,515-2,989) | 4.8  (-4.3-15.6) | 2.6  (2.6-2.7) | 1.5  (1.3-1.6) | -44.7  (-49.6--39.3) |
| B.2.2 - Ischemic heart disease | 127,107  (124,238-130,354) | 196,064  (187,695-206,243) | 54.3  (47-61.8) | 209.4  (204.6-214.6) | 117.6  (112.7-124) | -43.9  (-46.3--41) |
| B.2.3 - Cerebrovascular disease | 101,382  (97,582-103,993) | 144,080  (138,010-154,971) | 41.5  (35.1-58.6) | 162.9  (158.2-166.9) | 88  (84.2-94.8) | -46.2  (-48.6--40.4) |
| B.2.3.1 - Ischemic stroke | 31,285  (29,135-32,383) | 47,370  (44,937-52,424) | 50.1  (42.9-77.5) | 61.9  (57.7-64.1) | 31  (29.4-34.3) | -50.3  (-52.8--41.9) |
| B.2.3.2 - Hemorrhagic stroke | 70,097  (68,090-72,253) | 96,710  (92,217-103,494) | 37.5  (30.9-51.4) | 100.9  (98.1-104.1) | 57  (54.3-61.1) | -43.7  (-46.4--38.6) |
| B.2.4 - Hypertensive heart disease | 11,134  (10,349-11,749) | 24,037  (21,133-25,698) | 116.3  (97.4-130.7) | 18.2  (17-19.4) | 14.9  (13-15.9) | -18  (-26.6--12.1) |
| B.2.5 - Cardiomyopathy and myocarditis | 10,335  (8,866-10,843) | 19,356  (17,234-21,436) | 86.5  (73.6-108.4) | 14  (12-14.7) | 11.3  (10.1-12.5) | -19.7  (-25.5--9.8) |
| B.2.6 - Atrial fibrillation and flutter | 1,083  (824-1,398) | 4,050  (3,105-5,214) | 274  (251.3-300.4) | 2.7  (2-3.6) | 2.8  (2.1-3.6) | 2.7  (-2.4-8.8) |
| B.2.7 - Aortic aneurysm | 3,416  (3,287-3,554) | 9,952  (9,195-10,752) | 191.5  (168.2-215.1) | 5  (4.8-5.2) | 5.8  (5.4-6.3) | 16.9  (7.4-26.8) |
| B.2.8 - Peripheral vascular disease | 488  (460-522) | 2,592  (2,335-2,939) | 427.9  (375.1-497) | 0.9  (0.8-1) | 1.6  (1.5-1.9) | 81.4  (61.4-107.3) |
| B.2.9 - Endocarditis | 1,110  (946-1,525) | 2,556  (1,746-2,894) | 142.7  (59.6-165.3) | 1.2  (1-1.7) | 1.4  (1-1.6) | 25.3  (-19.5-36.6) |
| B.2.10 - Other cardiovascular and circulatory diseases | 8,971  (8,709-9,258) | 18,628  (17,624-19,841) | 107.4  (95.7-121.9) | 12.7  (12.3-13.1) | 11.1  (10.5-11.9) | -12.3  (-17.5--5.8) |
| **B.3 - Chronic respiratory diseases** | 41,272  (40,138-42,445) | 79,651  (76,015-83,698) | 92.8  (83.3-103.1) | 69.9  (67.8-71.9) | 49.7  (47.3-52.3) | -28.9  (-32.6--25.1) |
| B.3.1 - Chronic obstructive pulmonary disease | 36,712  (35,556-37,777) | 70,809  (67,375-74,758) | 92.5  (82.5-104.8) | 64.5  (62.4-66.3) | 44.5  (42.3-47.1) | -31.1  (-34.8--26.8) |
| B.3.2 - Pneumoconiosis | 156  (142-190) | 503  (425-557) | 236.7  (153.7-278.7) | 0.2  (0.2-0.3) | 0.3  (0.2-0.3) | 47.7  (8.3-65) |
| B.3.2.1 - Silicosis | 97  (89-111) | 260  (229-289) | 171.8  (125.3-211.6) | 0.1  (0.1-0.1) | 0.1  (0.1-0.2) | 22.8  (-0.9-41.3) |
| B.3.2.2 - Asbestosis | 12  (10-19) | 68  (53-80) | 512.1  (230.4-633.9) | 0  (0-0) | 0  (0-0) | 176  (44-237.3) |
| B.3.2.3 - Coal workers pneumoconiosis | 14  (12-19) | 60  (44-69) | 368.2  (199.1-447.5) | 0  (0-0) | 0  (0-0) | 100.1  (18.9-135) |
| B.3.2.4 - Other pneumoconiosis | 33 (30-41) | 115 (87-131) | 275.5 (144.8-323.4) | 0 (0-0.1) | 0.1 (0-0.1) | 45.6 (-7.6-65.1) |
| B.3.3 - Asthma | 2,800  (2,666-3,018) | 3,997  (3,688-4,417) | 42.7  (31.8-55.2) | 3.5  (3.3-3.8) | 2.3  (2.1-2.6) | -33.5  (-39--27.7) |
| B.3.4 - Interstitial lung disease and pulmonary sarcoidosis | 769  (662-999) | 3,350  (2,155-3,914) | 349.2  (135.5-411.8) | 1.1  (1-1.5) | 2  (1.3-2.4) | 90.4  (-6-118) |
| B.3.5 - Other chronic respiratory diseases | 835  (699-978) | 992  (713-1,133) | 22  (-12.9-38.5) | 0.6  (0.5-0.8) | 0.6  (0.4-0.6) | -3.3  (-35.8-8.7) |
| **B.4 - Cirrhosis and other chronic liver diseases** | 22,783  (21,290-23,771) | 36,607  (34,172-39,284) | 60.5  (50-72.7) | 25.7  (24.1-26.9) | 18.9  (17.6-20.3) | -26.7  (-31.2--21.3) |
| B.4.1 - Cirrhosis and other chronic liver diseases due to hepatitis B | 4,145  (3,759-4,529) | 6,289  (5,663-6,953) | 51.6  (41.6-63.1) | 5.1  (4.6-5.5) | 3.3  (3-3.7) | -34.6  (-38.6--29.6) |
| B.4.2 - Cirrhosis and other chronic liver diseases due to hepatitis C | 4,716  (4,356-5,053) | 7,286  (6,670-7,939) | 54.3  (43.8-66.4) | 5.6  (5.2-6) | 3.8  (3.5-4.1) | -32.2  (-36.4--27) |
| B.4.3 - Cirrhosis and other chronic liver diseases due to alcohol use | 10,707  (9,975-11,312) | 18,924  (17,540-20,344) | 76.6  (64.3-90.1) | 11.4  (10.7-12.1) | 9.5  (8.8-10.2) | -16.9  (-22.5--10.8) |
| B.4.4 - Cirrhosis and other chronic liver diseases due to other causes | 3,214  (2,954-3,425) | 4,108  (3,793-4,568) | 27.2  (19.8-40.2) | 3.6  (3.3-3.9) | 2.3  (2.1-2.5) | -38.3  (-41.7--31.8) |
| **B.5 - Digestive diseases** | 17,711  (16,770-18,591) | 33,267  (28,455-35,152) | 88.9  (62.3-99.3) | 25.3  (24.1-26.5) | 19.5  (16.6-20.7) | -22.4  (-33.6--18.2) |
| B.5.1 - Peptic ulcer disease | 4,531  (4,157-4,807) | 4,744  (4,259-5,491) | 3.2  (-5.9-21.2) | 6.6  (6.1-7.1) | 2.8  (2.5-3.2) | -58.6  (-62.4--51.4) |
| B.5.2 - Gastritis and duodenitis | 327  (273-420) | 693  (506-823) | 114.4  (71.2-145.1) | 0.5  (0.4-0.6) | 0.4  (0.3-0.5) | -14.4  (-32--1.7) |
| B.5.3 - Appendicitis | 871  (812-1,106) | 1,271  (980-1,410) | 51.2  (1.7-64.3) | 0.9  (0.8-1.1) | 0.7  (0.5-0.8) | -22.1  (-46.9--14.7) |
| B.5.4 - Paralytic ileus and intestinal obstruction | 2,560  (2,172-2,929) | 5,149  (4,038-5,734) | 102.3  (69.1-120.9) | 3.8  (3.2-4.4) | 3.1  (2.4-3.5) | -17  (-31.4--8.5) |
| B.5.5 - Inguinal, femoral, and abdominal hernia | 847  (671-1,149) | 1,606  (1,044-1,861) | 98.2  (38.4-123.3) | 1.3  (1.1-1.8) | 1  (0.6-1.1) | -23.7  (-47.9--13.6) |
| B.5.6 - Inflammatory bowel disease | 440  (376-471) | 934  (730-1,031) | 112.9  (77.8-134.6) | 0.5  (0.5-0.6) | 0.5  (0.4-0.6) | -4.4  (-20.7-5.9) |
| B.5.7 - Vascular intestinal disorders | 1,892  (1,717-2,009) | 3,810  (3,267-4,205) | 101.4  (83.5-119.9) | 3  (2.7-3.2) | 2.3  (2-2.6) | -24.1  (-31--16.6) |
| B.5.8 - Gallbladder and biliary diseases | 2,265  (2,103-2,414) | 5,543  (4,439-6,145) | 147.1  (97.1-170.2) | 3.5  (3.3-3.8) | 3.4  (2.7-3.7) | -4.5  (-23.3-5.5) |
| B.5.9 - Pancreatitis | 2,057  (1,866-2,201) | 4,729  (4,163-5,271) | 130.6  (105.1-151.7) | 2.3  (2.1-2.5) | 2.5  (2.2-2.8) | 8.6  (-4.5-19.2) |
| B.5.10 - Other digestive diseases | 1,921  (1,620-2,403) | 4,788  (3,688-5,486) | 152.5  (99.5-173.2) | 2.7  (2.3-3.3) | 2.8  (2.2-3.3) | 6.3  (-15.1-15.4) |
| **B.6 - Neurological disorders** | 19,714  (16,924-22,399) | 63,396  (53,548-73,278) | 221.4  (205.7-237.5) | 43.2  (36-50.3) | 42.2  (35.3-49.1) | -2.5  (-6-1.4) |
| B.6.1 - Alzheimer disease and other dementias | 16,012  (13,213-18,672) | 54,521  (44,734-64,521) | 240.3  (223.8-257.8) | 39.4  (32.2-46.5) | 37.1  (30.3-44) | -5.9  (-9.5--1.8) |
| B.6.2 - Parkinson disease | 510  (488-534) | 3,337  (2,999-3,656) | 556.8  (466.1-618.8) | 1  (0.9-1.1) | 2.2  (2-2.4) | 122  (88.5-144.9) |
| B.6.3 - Epilepsy | 1,816  (1,738-1,895) | 2,463  (2,326-2,614) | 35.3  (26-47.1) | 1.4  (1.3-1.4) | 1.2  (1.1-1.3) | -11.5  (-17--4.5) |
| B.6.4 - Multiple sclerosis | 145  (136-185) | 389  (345-436) | 173.1  (121.8-197.1) | 0.2  (0.2-0.2) | 0.2  (0.2-0.2) | 18.5  (-2.5-29.2) |
| B.6.8 - Motor neuron disease | 326  (312-340) | 1,064  (966-1,153) | 228.9  (190.4-256.9) | 0.4  (0.3-0.4) | 0.6  (0.5-0.6) | 60.8  (39.6-74.6) |
| B.6.8 - Other neurological disorders | 906  (876-942) | 1,623  (1,528-1,736) | 79  (67.7-91.2) | 0.9  (0.9-1) | 0.9  (0.8-0.9) | -8.1  (-13.8--1.1) |
| **B.7 - Mental and substance use disorders** | 5,079 (4,837-5,385) | 11,037 (10,308-11,828) | 117.7 (100.2-133.5) | 4.6 (4.4-4.9) | 5.3 (4.9-5.6) | 14.4 (5.4-22.5) |
| B.7.1 - Schizophrenia | 87  (82-91) | 380  (320-418) | 340.4  (284.9-390.8) | 0.1  (0.1-0.1) | 0.2  (0.2-0.2) | 106.3  (79.3-129.9) |
| B.7.2 - Alcohol use disorders | 4,431  (4,211-4,661) | 9,089  (8,486-9,733) | 105.5  (87.7-121.4) | 4.1  (3.9-4.3) | 4.3  (4-4.6) | 6.8  (-2.2-15.1) |
| B.7.3 - Drug use disorders | 546  (506-695) | 1,534  (1,340-1,920) | 183.7  (141-212.6) | 0.5  (0.4-0.6) | 0.7  (0.7-0.9) | 63.2  (37.6-78.9) |
| B.7.3.1 - Opioid use disorders | 458  (423-552) | 914  (793-1,239) | 98.1  (72-147.3) | 0.4  (0.3-0.5) | 0.4  (0.4-0.6) | 21.8  (6-41.6) |
| B.7.3.2 - Cocaine use disorders | 18  (13-56) | 236  (142-305) | 1532.2  (176.7-1895.9) | 0  (0-0.1) | 0.1  (0.1-0.1) | 691.6  (44-857.6) |
| B.7.3.3 - Amphetamine use disorders | 2  (2-4) | 44  (33-61) | 1970.3  (1287.4-2451.3) | 0  (0-0) | 0  (0-0) | 967.1  (598-1205.9) |
| B.7.3.5 - Other drug use disorders | 68  (61-103) | 341  (290-415) | 413.2  (237.6-499.8) | 0.1  (0.1-0.1) | 0.2  (0.1-0.2) | 121.5  (51.8-157.7) |
| B.7.7 - Eating disorders | 15  (11-32) | 33  (20-49) | 168.7  (50-209.2) | 0  (0-0) | 0  (0-0) | 81.4  (-0.5-107.8) |
| B.7.7.1 - Anorexia nervosa | 15  (11-30) | 31  (18-44) | 168.9  (39.5-209) | 0  (0-0) | 0  (0-0) | 83.4  (-6.1-110.1) |
| B.7.7.2 - Bulimia nervosa | 1  (0-2) | 2  (1-6) | 209.5  (145.4-310.1) | 0  (0-0) | 0  (0-0) | 68.7  (32.8-124.4) |
| **B.8 - Diabetes, urogenital, blood, and endocrine diseases** | 45,922  (44,606-48,180) | 124,066  (117,671-130,022) | 170.8  (155.6-182.7) | 65.9  (63.9-69.2) | 73.9  (70-77.4) | 12.6  (6.2-17.3) |
| B.8.1 - Diabetes mellitus | 23,802  (23,109-24,527) | 62,466  (59,420-65,474) | 162.6  (146.4-177.3) | 35.9  (34.8-37) | 37.5  (35.6-39.3) | 4.5  (-1.7-10.3) |
| B.8.2 - Acute glomerulonephritis | 247  (128-284) | 67  (59-94) | -75.7  (-78.2--38.4) | 0.2  (0.1-0.2) | 0  (0-0.1) | -82.4  (-84.1--57.6) |
| B.8.3 - Chronic kidney disease | 14,788  (13,821-16,835) | 36,337  (33,760-41,391) | 145.7  (130.4-161) | 20.6  (19.5-23.6) | 21.4  (19.9-24.2) | 4.2  (-2.9-10.3) |
| B.8.3.1 - Chronic kidney disease due to diabetes mellitus | 6,992  (6,477-8,087) | 21,519  (19,905-24,480) | 208.1  (187.9-227.1) | 10  (9.3-11.7) | 12.5  (11.5-14.1) | 24.4  (15.9-31.7) |
| B.8.3.2 - Chronic kidney disease due to hypertension | 6,168  (5,722-7,023) | 12,933  (11,783-14,853) | 109.8  (94.8-124.1) | 8.6  (8-9.8) | 7.8  (7.1-8.9) | -8.6  (-15--2.8) |
| B.8.3.3 - Chronic kidney disease due to glomerulonephritis | 1,541  (1,358-1,761) | 1,705  (1,506-2,002) | 10.1  (0.8-22.6) | 1.9  (1.6-2.2) | 1  (0.9-1.2) | -45.5  (-50.3--40.3) |
| B.8.3.4 - Chronic kidney disease due to other causes | 88  (76-101) | 181  (158-214) | 104.9  (86.4-128.8) | 0.1  (0.1-0.1) | 0.1  (0.1-0.1) | -1.5  (-8.7-5.6) |
| B.8.4 - Urinary diseases and male infertility | 3,487  (3,058-3,840) | 14,055  (8,809-15,750) | 316.1  (154.6-350.3) | 5.4  (4.6-5.9) | 8.8  (5.5-9.9) | 68.6  (2.6-83.2) |
| B.8.4.1 - Interstitial nephritis and urinary tract infections | 2,986  (2,584-3,265) | 12,663  (7,342-14,294) | 337.2  (155.5-374.8) | 4.7  (4-5.1) | 8  (4.6-9.1) | 77.3  (3.6-93.3) |
| B.8.4.2 - Urolithiasis | 90  (76-129) | 318  (151-385) | 299.2  (67.6-347.3) | 0.1  (0.1-0.2) | 0.2  (0.1-0.2) | 73.8  (-28.5-96) |
| B.8.4.5 - Other urinary diseases | 410  (334-554) | 1,074  (812-1,438) | 162.7  (122.9-200.8) | 0.6  (0.5-0.8) | 0.6  (0.5-0.8) | 0.8  (-12-22) |
| B.8.5 - Gynecological diseases | 86  (75-109) | 170  (91-200) | 106.2  (9.6-141.8) | 0.1  (0.1-0.1) | 0.1  (0-0.1) | -0.6  (-47.9-16.7) |
| B.8.5.1 - Uterine fibroids | 21  (17-32) | 52  (22-67) | 170.9  (-4.4-238.5) | 0  (0-0) | 0  (0-0) | 25.1  (-55.1-57.4) |
| B.8.5.2 - Polycystic ovarian syndrome | 3  (2-5) | 1  (1-2) | -65.1  (-74.5--44.7) | 0  (0-0) | 0  (0-0) | -83  (-87.5--72.7) |
| B.8.5.4 - Endometriosis | 2  (2-3) | 8  (5-14) | 238.6  (123.1-440.9) | 0  (0-0) | 0  (0-0) | 96.5  (30.2-214.6) |
| B.8.5.5 - Genital prolapse | 10 (9-16) | 29 (16-46) | 186.9 (27.8-319.1) | 0 (0-0) | 0 (0-0) | 4.3 (-53.1-53.4) |
| B.8.5.7 - Other gynecological diseases | 49  (36-56) | 79  (41-98) | 65.8  (-4.5-108.2) | 0  (0-0.1) | 0  (0-0.1) | -13.8  (-50.9-7.7) |
| B.8.6 - Hemoglobinopathies and hemolytic anemias | 1,489  (1,361-1,739) | 2,122  (1,879-2,414) | 43.3  (29.7-53.4) | 1.6  (1.5-1.8) | 1.2  (1-1.3) | -27.3  (-34.1--22.3) |
| B.8.6.1 - Thalassemias | 147  (129-179) | 115  (94-133) | -20.6  (-38--6.6) | 0.1  (0.1-0.1) | 0.1  (0.1-0.1) | -19.8  (-35.7--6.5) |
| B.8.6.2 - Sickle cell disorders | 301  (272-350) | 419  (353-464) | 40.1  (21.7-53.1) | 0.2  (0.2-0.2) | 0.2  (0.2-0.2) | 6  (-6.7-16.4) |
| B.8.6.3 - G6PD deficiency | 283  (247-345) | 498  (408-575) | 78.4  (47.8-94.9) | 0.2  (0.2-0.3) | 0.2  (0.2-0.3) | 9.6  (-8.3-19.6) |
| B.8.6.4 - Other hemoglobinopathies and hemolytic anemias | 759  (687-860) | 1,090  (965-1,301) | 44.5  (29-56.8) | 1.1  (1-1.3) | 0.7  (0.6-0.8) | -40.8  (-47.6--35) |
| B.8.7 - Endocrine, metabolic, blood, and immune disorders | 2,023  (1,939-2,115) | 8,850  (8,205-9,617) | 338.9  (302.1-373.9) | 2.2  (2.1-2.3) | 4.9  (4.6-5.4) | 128.8  (109.9-147.6) |
| **B.9 - Musculoskeletal disorders** | 1,454  (1,264-1,544) | 3,553  (3,277-4,182) | 142.4  (125.6-180) | 1.6  (1.4-1.7) | 1.9  (1.8-2.3) | 19  (10.5-38.9) |
| B.9.1 - Rheumatoid arthritis | 253  (198-303) | 592  (428-692) | 135.2  (98.9-160.2) | 0.4  (0.3-0.4) | 0.3  (0.2-0.4) | -6.5  (-20.1-3.7) |
| B.9.5 - Other musculoskeletal disorders | 1,201  (1,039-1,295) | 2,961  (2,725-3,566) | 144  (126.5-189.8) | 1.2  (1.1-1.3) | 1.6  (1.4-1.9) | 26.7  (17.4-52) |
| **B.10 - Other non-communicable diseases** | 15,988  (13,668-22,657) | 17,519  (13,174-19,780) | 17.3  (-36.9-37.6) | 10  (8.7-13.8) | 10.4  (7.8-11.8) | 7.3  (-39.1-29.1) |
| B.10.1 - Congenital anomalies | 13,783  (11,532-19,994) | 11,495  (7,902-13,349) | -11.6  (-61.3-9.4) | 7.3  (6.1-10.5) | 6.8  (4.6-7.9) | -0.7  (-57.2-22.9) |
| B.10.1.1 - Neural tube defects | 1,419  (1,162-2,086) | 714  (461-871) | -45.3  (-79.7--30.1) | 0.7  (0.6-1.1) | 0.4  (0.3-0.5) | -35.6  (-76.1--17.4) |
| B.10.1.2 - Congenital heart anomalies | 7,427  (6,196-10,656) | 4,837  (3,440-5,641) | -30.5  (-68.4--13.1) | 3.9  (3.3-5.6) | 2.8  (2-3.3) | -23.3  (-66.1--3.6) |
| B.10.1.3 - Cleft lip and cleft palate | 50  (38-81) | 26  (16-35) | -43.2  (-77.8--17.5) | 0  (0-0) | 0  (0-0) | -32.2  (-73.5--1.5) |
| B.10.1.4 - Down syndrome | 401  (311-782) | 459  (191-595) | 26.7  (-73.9-78.5) | 0.2  (0.2-0.4) | 0.3  (0.1-0.3) | 31.3  (-73.1-85.3) |
| B.10.1.7 - Other chromosomal abnormalities | 108  (84-215) | 344  (105-454) | 276  (-50.3-392.8) | 0.1  (0-0.1) | 0.2  (0.1-0.3) | 329.9  (-43.2-464.5) |
| B.10.1.8 - Other congenital anomalies | 4,378  (3,701-6,086) | 5,115  (3,673-5,992) | 22.2  (-38.8-52.9) | 2.3  (2-3.2) | 3.1  (2.2-3.6) | 37.4  (-31.8-72.4) |
| B.10.2 - Skin and subcutaneous diseases | 2,001  (1,237-3,180) | 5,766  (3,218-8,508) | 193.7  (143.9-222.2) | 2.7  (1.7-4.3) | 3.5  (1.9-5.2) | 36  (5.1-52.1) |
| B.10.2.3 - Cellulitis | 179  (110-305) | 631  (263-861) | 299  (86.7-360.4) | 0.2  (0.1-0.4) | 0.4  (0.2-0.5) | 104.6  (-11.4-139.9) |
| B.10.2.4 - Pyoderma | 1,229  (737-1,927) | 3,303  (1,874-5,101) | 170.1  (139.7-193.8) | 1.4  (0.9-2.3) | 2  (1.1-3) | 39.3  (13.1-55.1) |
| B.10.2.12 - Decubitus ulcer | 465  (267-736) | 1,591  (745-2,227) | 257.7  (154.1-312.8) | 0.9  (0.5-1.4) | 1  (0.5-1.4) | 21.7  (-12.7-44) |
| B.10.2.13 - Other skin and subcutaneous diseases | 128  (80-212) | 240  (141-382) | 86.1  (61.3-138.1) | 0.1  (0.1-0.2) | 0.1  (0.1-0.2) | -5.5  (-21.6-20.2) |
| B.10.5 - Sudden infant death syndrome | 204  (167-299) | 258  (194-308) | 35.5  (-29.4-66.9) | 0.1  (0.1-0.2) | 0.2  (0.1-0.2) | 60.9  (-16.1-98.1) |
| **C - Injuries** | 134,931  (131,104-138,852) | 168,018  (159,904-177,046) | 24.5  (18.4-31.2) | 105.1  (101.7-108.1) | 81.2  (77.4-85.4) | -22.8  (-26.5--18.6) |
| **C.1 - Transport injuries** | 48,618  (46,835-50,494) | 54,601  (51,381-60,111) | 11.8  (5.5-27.2) | 37.3  (36-38.6) | 25.9  (24.4-28.4) | -30.9  (-34.9--21.3) |
| C.1.1 - Road injuries | 48,060 (46,231-49,893) | 52,326 (49,298-57,696) | 8.4 (2.3-24) | 36.9 (35.6-38.2) | 24.8 (23.4-27.3) | -33 (-36.9--23.2) |
| C.1.1.1 - Pedestrian road injuries | 25,135  (23,482-27,565) | 21,444  (19,508-24,086) | -14.9  (-23.6--3) | 20.1  (18.7-21.9) | 10.6  (9.7-11.8) | -47.7  (-52.6--40.6) |
| C.1.1.2 - Cyclist road injuries | 1,012  (934-1,175) | 2,148  (1,932-2,544) | 111.7  (88.1-144.4) | 0.8  (0.7-0.9) | 1  (0.9-1.2) | 33.4  (19.1-53.3) |
| C.1.1.3 - Motorcyclist road injuries | 5,817  (4,983-7,152) | 13,175  (9,098-16,484) | 131.7  (36.1-166.2) | 3.9  (3.4-4.8) | 5.9  (4.1-7.4) | 53.3  (-9-77.4) |
| C.1.1.4 - Motor vehicle road injuries | 15,912  (13,285-17,667) | 14,767  (13,271-18,735) | -9.3  (-17-17) | 11.9  (10-13.3) | 6.9  (6.2-8.8) | -43  (-47.7--26.9) |
| C.1.1.5 - Other road injuries | 184  (151-355) | 793  (405-1,033) | 391.8  (51.1-520.8) | 0.1  (0.1-0.3) | 0.4  (0.2-0.5) | 208  (-5.7-288.9) |
| C.1.2 - Other transport injuries | 559  (504-656) | 2,275  (1,839-2,582) | 312.4  (221.4-371.7) | 0.4  (0.4-0.5) | 1.1  (0.9-1.2) | 159.8  (102.2-196.6) |
| **C.2 - Unintentional injuries** | 34,975  (32,391-36,301) | 38,144  (35,781-40,384) | 8.8  (3.1-17.3) | 31.2  (28.3-32.5) | 20.9  (19.6-22.2) | -33.2  (-36.8--27) |
| C.2.1 - Falls | 7,221  (6,210-7,578) | 15,658  (12,710-16,955) | 117.1  (90.7-137.2) | 9.8  (8.5-10.3) | 9.3  (7.5-10.1) | -4.6  (-17.4-3.5) |
| C.2.2 - Drowning | 10,287  (9,780-11,139) | 6,856  (6,402-7,538) | -33.5  (-38.3--27.5) | 6.5  (6.2-7) | 3.3  (3.1-3.6) | -49.4  (-52.9--44.9) |
| C.2.3 - Fire, heat, and hot substances | 2,939  (2,233-3,191) | 1,990  (1,797-2,553) | -36.6  (-41.2--5) | 2.6  (2-2.8) | 1.1  (1-1.4) | -60.7  (-63.4--41.1) |
| C.2.4 - Poisonings | 656  (489-720) | 439  (393-600) | -35.6  (-40.6--10.9) | 0.5  (0.4-0.5) | 0.2  (0.2-0.3) | -53.1  (-56.5--38.8) |
| C.2.5 - Exposure to mechanical forces | 3,877  (3,282-4,242) | 2,807  (2,459-3,439) | -28.8  (-34.2--12.2) | 2.9  (2.4-3.1) | 1.4  (1.2-1.7) | -52.3  (-55.4--41.1) |
| C.2.5.1 - Unintentional firearm injuries | 1,487  (839-1,651) | 940  (764-1,261) | -39.7  (-46.4--3.3) | 1.2  (0.7-1.3) | 0.5  (0.4-0.6) | -63.2  (-67.1--38.9) |
| C.2.5.2 - Unintentional suffocation | 660  (491-726) | 263  (228-369) | -62.4  (-66.8--26.2) | 0.3  (0.3-0.4) | 0.1  (0.1-0.2) | -61.4  (-66.1--23.6) |
| C.2.5.3 - Other exposure to mechanical forces | 1,729  (1,561-2,184) | 1,604  (1,314-2,007) | -6.8  (-25-7.2) | 1.3  (1.2-1.7) | 0.8  (0.6-1) | -40  (-51.9--30.8) |
| C.2.6 - Adverse effects of medical treatment | 2,399  (1,801-3,049) | 2,840  (2,145-3,744) | 18.3  (10.9-26.3) | 2.6  (1.9-3.2) | 1.6  (1.2-2.1) | -37.9  (-42--33.5) |
| C.2.7 - Animal contact | 653  (583-727) | 615  (550-875) | -8.7  (-14.8-24.5) | 0.6  (0.5-0.6) | 0.3  (0.3-0.5) | -43.1  (-47.2--25.8) |
| C.2.7.1 - Venomous animal contact | 495  (429-541) | 387  (348-528) | -23.8  (-29-1.5) | 0.4  (0.4-0.5) | 0.2  (0.2-0.3) | -52.3  (-55.7--39.2) |
| C.2.7.2 - Non-venomous animal contact | 158  (146-193) | 227  (197-361) | 40  (23.9-88.3) | 0.1  (0.1-0.2) | 0.1  (0.1-0.2) | -14.6  (-24.3-11.1) |
| C.2.8 - Foreign body | 3,562  (2,382-3,979) | 3,394  (2,497-3,814) | -6.4  (-17.7-32.5) | 3.1  (2-3.6) | 2  (1.4-2.2) | -38.9  (-44.8--7.7) |
| C.2.8.1 - Pulmonary aspiration and foreign body in airway | 3,139  (1,954-3,539) | 3,013  (2,099-3,419) | -5.6  (-18.9-43.2) | 2.7  (1.6-3.2) | 1.8  (1.2-2) | -37.9  (-44.5-2.2) |
| C.2.8.3 - Foreign body in other body part | 423  (253-459) | 381  (296-424) | -11.4  (-19-26.3) | 0.4  (0.2-0.4) | 0.2  (0.2-0.2) | -46.4  (-51.7--28) |
| C.2.9 - Other unintentional injuries | 2,803  (2,679-2,943) | 3,223  (2,857-3,640) | 15  (1.3-28.6) | 2  (1.9-2.1) | 1.6  (1.4-1.8) | -22.7  (-31.9--13.7) |
| C.2.10 - Environmental heat and cold exposure | 577  (288-663) | 323  (271-541) | -53.3  (-57.7-21.9) | 0.7  (0.3-0.8) | 0.2  (0.1-0.3) | -79.8  (-81.8--39.8) |
| **C.3 - Self-harm and interpersonal violence** | 51,048  (48,599-55,573) | 75,273  (69,169-80,229) | 48.2  (30-57.5) | 36.4  (34.8-39.8) | 34.3  (31.7-36.6) | -5.1  (-16.9-0.8) |
| C.3.1 - Self-harm | 9,882  (9,232-10,550) | 14,036  (12,973-16,861) | 41.3  (30-63.8) | 8.1  (7.5-8.6) | 6.6  (6.1-7.9) | -19.3  (-25.4--4.6) |
| C.3.2 - Interpersonal violence | 41,166 (38,893-46,017) | 61,237 (53,401-65,653) | 50.1 (22.8-59.9) | 28.3 (26.9-32.1) | 27.8 (24.3-29.8) | -0.9 (-19.5-5.5) |
| C.3.2.1 - Assault by firearm | 23,022  (21,245-27,908) | 42,744  (34,739-46,161) | 91  (36.1-108.3) | 15.5  (14.4-18.7) | 19.3  (15.7-20.8) | 27.5  (-9.3-38.9) |
| C.3.2.2 - Assault by sharp object | 7,352  (6,824-9,240) | 10,874  (9,850-12,002) | 49.3  (19.9-66.1) | 5.3  (4.9-6.6) | 4.9  (4.5-5.5) | -5  (-22.6-5.4) |
| C.3.2.3 - Assault by other means | 10,792  (6,787-12,012) | 7,619  (6,331-9,199) | -32  (-39-2.3) | 7.6  (5-8.4) | 3.6  (3-4.3) | -54.2  (-58.7--34.1) |
| **C.4 - Forces of nature, war, and legal intervention** | 290  (85-648) | NA  (NA-NA) | -100  (-100--100) | 0.2  (0.1-0.5) | NA  (NA-NA) | -100  (-100--100) |
| C.4.1 - Exposure to forces of nature | 290  (85-648) | NA  (NA-NA) | -100  (-100--100) | 0.2  (0.1-0.5) | NA  (NA-NA) | -100  (-100--100) |
| C.4.2 - Collective violence and legal intervention | NA  (NA-NA) | NA  (NA-NA) | NA  (NA-NA) | NA  (NA-NA) | NA  (NA-NA) | NA  (NA-NA) |

| **Additional file 2: Table B- Number of deaths and age-standardized rates for 249 causes of death, male. Brazil ,1990 and 2015.** | | | | | | |
| --- | --- | --- | --- | --- | --- | --- |
|  |  |  |  |  |  |  |
| **Causes of death** | **Number of deaths** | | | **Age-standardized death rates (per 100,000)** | | |
|  | **1990** | **2015** | **% change*** | **1990** | **2015** | **% change*** |
| **All causes** | **534,116** | **772,909** | **44.7** | **1399.6** | **1008.5** | **-27.9** |
| **A - Communicable, maternal, neonatal, and nutritional diseases** | **130,049** | **89,230** | **-31.4** | **210.6** | **115.0** | **-45.4** |
| **A.1 - HIV/AIDS and tuberculosis** | **9,893** | **19,856** | **100.7** | **19.9** | **19.6** | **-1.7** |
| A.1.1 – Tuberculosis | 5,373 | 4,185 | -22.1 | 12.6 | 4.8 | -62.4 |
| A.1.2 - HIV/AIDS | 4,520 | 15,672 | 246.7 | 7.2 | 14.8 | 104.2 |
| A.1.2.1 - HIV/AIDS – Tuberculosis | 1,179 | 1,456 | 23.5 | 1.9 | 1.4 | -27.5 |
| A.1.2.2 - HIV/AIDS resulting in other diseases | 3,341 | 14,216 | 325.5 | 5.3 | 13.4 | 151.0 |
| **A.2 - Diarrhea, lower respiratory, and other common infectious diseases** | **65,688** | **43,985** | **-33.0** | **119.2** | **63.4** | **-46.8** |
| A.2.1 - Diarrheal diseases | 25,169 | 3,213 | -87.2 | 33.1 | 4.4 | -86.6 |
| A.2.2 - Intestinal infectious diseases | 74 | 27 | -63.2 | 0.1 | 0.0 | -71.8 |
| A.2.2.1 - Typhoid fever | 30 | 22 | -27.1 | 0.1 | 0.0 | -54.1 |
| A.2.2.2 - Paratyphoid fever | 0 | 1 | 291.0 | 0.0 | 0.0 | 158.4 |
| A.2.2.3 - Other intestinal infectious diseases | 44 | 5 | -89.3 | 0.1 | 0.0 | -89.7 |
| A.2.3 - Lower respiratory infections | 32,875 | 38,586 | 17.4 | 76.6 | 56.6 | -26.1 |
| A.2.4 - Upper respiratory infections | 28 | 27 | -5.6 | 0.1 | 0.0 | -46.2 |
| A.2.5 - Otitis media | 119 | 26 | -78.2 | 0.1 | 0.0 | -80.0 |
| A.2.6 – Meningitis | 4,563 | 1,650 | -63.8 | 5.5 | 1.8 | -67.2 |
| A.2.6.1 - Pneumococcal meningitis | 830 | 255 | -69.3 | 1.0 | 0.3 | -71.2 |
| A.2.6.2 - H influenzae type B meningitis | 576 | 57 | -90.1 | 0.6 | 0.1 | -89.6 |
| A.2.6.3 - Meningococcal meningitis | 1,504 | 580 | -61.4 | 1.8 | 0.6 | -64.2 |
| A.2.6.4 - Other meningitis | 1,653 | 758 | -54.1 | 2.1 | 0.8 | -61.2 |
| A.2.7 – Encephalitis | 512 | 240 | -53.2 | 0.7 | 0.3 | -61.5 |
| A.2.8 – Diphtheria | 19 | 3 | -86.1 | 0.0 | 0.0 | -87.3 |
| A.2.9 - Whooping cough | 892 | 40 | -95.5 | 0.9 | 0.0 | -94.8 |
| A.2.10 – Tetanus | 727 | 150 | -79.4 | 1.2 | 0.2 | -86.4 |
| A.2.11 – Measles | 562 | 2 | -99.7 | 0.6 | 0.0 | -99.6 |
| A.2.12 - Varicella and herpes zoster | 146 | 21 | -85.8 | 0.3 | 0.0 | -91.8 |
| **A.3 - Neglected tropical diseases and malaria** | **5,955** | **4,301** | **-27.8** | **14.6** | **5.3** | **-63.5** |
| A.3.1 - Malaria | 489 | 40 | -91.9 | 0.6 | 0.0 | -93.8 |
| A.3.2 - Chagas disease | 4,415 | 3,397 | -23.1 | 11.8 | 4.3 | -63.1 |
| A.3.3 - Leishmaniasis | 224 | 311 | 39.2 | 0.4 | 0.3 | -13.8 |
| A.3.3.1 - Visceral leishmaniasis | 224 | 311 | 39.2 | 0.4 | 0.3 | -13.8 |
| A.3.4 - African trypanosomiasis | 0 | 0 | - | 0.0 | 0.0 | - |
| A.3.5 - Schistosomiasis | 559 | 161 | -71.2 | 1.4 | 0.2 | -86.7 |
| A.3.6 - Cysticercosis | 93 | 22 | -76.1 | 0.2 | 0.0 | -86.9 |
| A.3.7 - Cystic echinococcosis | 9 | 3 | -63.4 | 0.0 | 0.0 | -80.0 |
| A.3.11 - Dengue | 15 | 262 | 1597.4 | 0.0 | 0.3 | 980.9 |
| A.3.12 - Yellow fever | 12 | 5 | -62.5 | 0.0 | 0.0 | -71.7 |
| A.3.13 - Rabies | 42 | 3 | -92.1 | 0.1 | 0.0 | -93.8 |
| A.3.14 - Intestinal nematode infections | 20 | 10 | -51.8 | 0.0 | 0.0 | -54.7 |
| A.3.14.1 - Ascariasis | 20 | 10 | -51.8 | 0.0 | 0.0 | -54.7 |
| A.3.17 - Ebola | 0 | 0 | - | 0.0 | 0.0 | - |
| A.3.19 - Other neglected tropical diseases | 78 | 88 | 11.8 | 0.1 | 0.1 | -3.8 |
| **A.4 - Maternal disorders** | **0** | **0** | **-** | **0.0** | **0.0** | **-** |
| A.4.1 - Maternal hemorrhage | 0 | 0 | - | 0.0 | 0.0 | - |
| A.4.2 - Maternal sepsis and other maternal infections | 0 | 0 | - | 0.0 | 0.0 | - |
| A.4.3 - Maternal hypertensive disorders | 0 | 0 | - | 0.0 | 0.0 | - |
| A.4.4 - Maternal obstructed labor and uterine rupture | 0 | 0 | - | 0.0 | 0.0 | - |
| A.4.5 - Maternal abortion, miscarriage, and ectopic pregnancy | 0 | 0 | - | 0.0 | 0.0 | - |
| A.4.6 - Indirect maternal deaths | 0 | 0 | - | 0.0 | 0.0 | - |
| A.4.7 - Late maternal deaths | 0 | 0 | - | 0.0 | 0.0 | - |
| A.4.8 - Maternal deaths aggravated by HIV/AIDS | 0 | 0 | - | 0.0 | 0.0 | - |
| A.4.9 - Other maternal disorders | 0 | 0 | - | 0.0 | 0.0 | - |
| **A.5 - Neonatal disorders** | **38,489** | **14,072** | **-63.4** | **38.7** | **17.0** | **-56.2** |
| A.5.1 - Neonatal preterm birth complications | 22,980 | 5,415 | -76.4 | 23.1 | 6.5 | -71.7 |
| A.5.2 - Neonatal encephalopathy due to birth asphyxia and trauma | 7,734 | 3,313 | -57.2 | 7.8 | 4.0 | -48.6 |
| A.5.3 - Neonatal sepsis and other neonatal infections | 5,069 | 2,764 | -45.5 | 5.1 | 3.3 | -34.8 |
| A.5.4 - Hemolytic disease and other neonatal jaundice | 617 | 121 | -80.3 | 0.6 | 0.1 | -76.5 |
| A.5.5 - Other neonatal disorders | 2,088 | 2,459 | 17.7 | 2.1 | 3.0 | 41.0 |
| **A.6 - Nutritional deficiencies** | **7,380** | **5,049** | **-31.6** | **14.2** | **7.5** | **-46.8** |
| A.6.1 - Protein-energy malnutrition | 6,915 | 4,661 | -32.6 | 13.2 | 7.0 | -47.1 |
| A.6.2 - Iodine deficiency | 11 | 8 | -28.7 | 0.0 | 0.0 | -54.0 |
| A.6.4 - Iron-deficiency anemia | 10 | 15 | 48.3 | 0.0 | 0.0 | 19.8 |
| A.6.5 - Other nutritional deficiencies | 443 | 364 | -17.7 | 0.9 | 0.5 | -43.9 |
| **A.7 - Other communicable, maternal, neonatal, and nutritional diseases** | **2,645** | **1,967** | **-25.6** | **4.1** | **2.2** | **-46.6** |
| A.7.1 - Sexually transmitted diseases excluding HIV | 1,109 | 145 | -86.9 | 1.2 | 0.2 | -85.1 |
| A.7.1.1 - Syphilis | 1,105 | 142 | -87.2 | 1.2 | 0.2 | -85.3 |
| A.7.1.2 - Chlamydial infection | 0 | 0 | - | 0.0 | 0.0 | - |
| A.7.1.3 - Gonococcal infection | 3 | 2 | -15.4 | 0.0 | 0.0 | -60.3 |
| A.7.1.6 - Other sexually transmitted diseases | 1 | 1 | -21.7 | 0.0 | 0.0 | -62.5 |
| A.7.2 - Hepatitis | 644 | 786 | 21.9 | 1.2 | 0.9 | -30.4 |
| A.7.2.1 - Acute hepatitis A | 155 | 60 | -61.3 | 0.2 | 0.1 | -61.8 |
| A.7.2.2 - Acute hepatitis B | 465 | 688 | 47.7 | 1.0 | 0.8 | -25.8 |
| A.7.2.3 - Acute hepatitis C | 24 | 38 | 59.6 | 0.1 | 0.0 | -22.3 |
| A.7.2.4 - Acute hepatitis E | 0 | 0 | - | 0.0 | 0.0 | - |
| A.7.4 - Other infectious diseases | 892 | 1,036 | 16.2 | 1.7 | 1.1 | -31.6 |
| **B - Non-communicable diseases** | **296,200** | **547,437** | **84.8** | **1018.1** | **759.4** | **-25.4** |
| **B.1 - Neoplasms** | **56,877** | **128,980** | **126.8** | **182.3** | **172.1** | **-5.6** |
| B.1.1 - Lip and oral cavity cancer | 1,619 | 3,652 | 125.5 | 4.7 | 4.4 | -6.4 |
| B.1.2 - Nasopharynx cancer | 134 | 346 | 159.1 | 0.3 | 0.4 | 24.1 |
| B.1.3 - Other pharynx cancer | 1,248 | 2,879 | 130.7 | 3.4 | 3.3 | -2.8 |
| B.1.4 - Esophageal cancer | 4,030 | 8,523 | 111.5 | 12.2 | 10.5 | -14.1 |
| B.1.5 - Stomach cancer | 9,255 | 14,567 | 57.4 | 31.0 | 19.5 | -37.3 |
| B.1.6 - Colon and rectum cancer | 3,197 | 10,502 | 228.5 | 10.9 | 14.1 | 29.5 |
| B.1.7 - Liver cancer | 2,394 | 5,848 | 144.3 | 7.5 | 7.5 | -0.9 |
| B.1.7.1 - Liver cancer due to hepatitis B | 694 | 1,379 | 98.7 | 1.8 | 1.6 | -11.9 |
| B.1.7.2 - Liver cancer due to hepatitis C | 723 | 1,638 | 126.7 | 2.6 | 2.2 | -13.4 |
| B.1.7.3 - Liver cancer due to alcohol use | 715 | 2,320 | 224.7 | 2.3 | 3.0 | 28.8 |
| B.1.7.4 - Liver cancer due to other causes | 262 | 511 | 94.7 | 0.8 | 0.7 | -21.0 |
| B.1.8 - Gallbladder and biliary tract cancer | 819 | 1,489 | 81.8 | 2.9 | 2.0 | -29.8 |
| B.1.9 - Pancreatic cancer | 2,102 | 5,382 | 156.1 | 6.9 | 7.1 | 2.6 |
| B.1.10 - Larynx cancer | 2,045 | 4,164 | 103.7 | 6.0 | 5.1 | -15.3 |
| B.1.11 - Tracheal, bronchus, and lung cancer | 9,273 | 19,546 | 110.8 | 29.5 | 25.9 | -12.0 |
| B.1.12 - Malignant skin melanoma | 407 | 1,129 | 177.4 | 1.1 | 1.4 | 25.8 |
| B.1.13 - Non-melanoma skin cancer | 627 | 1,756 | 180.0 | 2.2 | 2.5 | 13.7 |
| B.1.13.1 - Non-melanoma skin cancer (squamous-cell carcinoma) | 627 | 1,756 | 180.0 | 2.2 | 2.5 | 13.7 |
| B.1.14 - Mesothelioma | 374 | 402 | 7.6 | 1.2 | 0.5 | -59.2 |
| B.1.15 - Breast cancer | 75 | 340 | 350.3 | 0.2 | 0.4 | 88.5 |
| B.1.16 - Cervical cancer | 0 | 0 | - | 0.0 | 0.0 | - |
| B.1.17 - Uterine cancer | 0 | 0 | - | 0.0 | 0.0 | - |
| B.1.18 - Ovarian cancer | 0 | 0 | - | 0.0 | 0.0 | - |
| B.1.19 - Prostate cancer | 6,454 | 21,130 | 227.4 | 29.8 | 33.4 | 12.2 |
| B.1.20 - Testicular cancer | 189 | 337 | 77.9 | 0.3 | 0.3 | 0.7 |
| B.1.21 - Kidney cancer | 745 | 2,384 | 220.2 | 2.1 | 3.0 | 44.8 |
| B.1.22 - Bladder cancer | 1,316 | 3,248 | 146.8 | 5.3 | 4.9 | -8.4 |
| B.1.23 - Brain and nervous system cancer | 1,591 | 4,422 | 178.0 | 3.4 | 5.0 | 48.4 |
| B.1.24 - Thyroid cancer | 180 | 411 | 127.5 | 0.6 | 0.5 | -5.7 |
| B.1.25 - Hodgkin lymphoma | 449 | 483 | 7.7 | 1.0 | 0.5 | -44.4 |
| B.1.26 - Non-Hodgkin lymphoma | 1,514 | 3,236 | 113.7 | 3.7 | 4.0 | 7.9 |
| B.1.27 - Multiple myeloma | 496 | 1,674 | 237.7 | 1.5 | 2.1 | 43.6 |
| B.1.28 - Leukemia | 2,986 | 5,240 | 75.5 | 6.4 | 6.5 | 0.5 |
| B.1.28.1.1 - Acute lymphoid leukemia | 1,077 | 1,387 | 28.8 | 1.6 | 1.5 | -8.6 |
| B.1.28.1.2 - Chronic lymphoid leukemia | 247 | 779 | 215.0 | 1.0 | 1.2 | 21.3 |
| B.1.28.2.1 - Acute myeloid leukemia | 1,194 | 2,456 | 105.7 | 2.6 | 3.0 | 13.9 |
| B.1.28.2.2 - Chronic myeloid leukemia | 468 | 618 | 32.1 | 1.2 | 0.8 | -32.5 |
| B.1.29 - Other neoplasms | 3,359 | 5,891 | 75.3 | 8.1 | 7.2 | -11.2 |
| **B.2 - Cardiovascular diseases** | **144,700** | **223,808** | **54.7** | **524.8** | **315.8** | **-39.8** |
| B.2.1 - Rheumatic heart disease | 1,218 | 1,176 | -3.4 | 2.9 | 1.4 | -50.2 |
| B.2.2 - Ischemic heart disease | 71,365 | 107,602 | 50.8 | 260.6 | 148.9 | -42.9 |
| B.2.3 - Cerebrovascular disease | 52,611 | 73,488 | 39.7 | 195.2 | 106.9 | -45.2 |
| B.2.3.1 - Ischemic stroke | 16,342 | 24,433 | 49.5 | 77.4 | 39.6 | -48.8 |
| B.2.3.2 - Hemorrhagic stroke | 36,269 | 49,054 | 35.3 | 117.8 | 67.3 | -42.8 |
| B.2.4 - Hypertensive heart disease | 5,167 | 10,822 | 109.5 | 19.3 | 16.0 | -17.0 |
| B.2.5 - Cardiomyopathy and myocarditis | 5,802 | 11,243 | 93.8 | 17.3 | 14.8 | -14.4 |
| B.2.6 - Atrial fibrillation and flutter | 539 | 1,979 | 267.4 | 3.6 | 3.6 | 0.0 |
| B.2.7 - Aortic aneurysm | 2,495 | 6,153 | 146.6 | 8.7 | 8.5 | -2.4 |
| B.2.8 - Peripheral vascular disease | 277 | 1,327 | 379.3 | 1.2 | 2.0 | 70.9 |
| B.2.9 - Endocarditis | 606 | 1,377 | 127.4 | 1.4 | 1.7 | 20.0 |
| B.2.10 - Other cardiovascular and circulatory diseases | 4,621 | 8,640 | 87.0 | 14.7 | 12.0 | -18.7 |
| **B.3 - Chronic respiratory diseases** | **24,114** | **45,255** | **87.7** | **100.9** | **69.4** | **-31.3** |
| B.3.1 - Chronic obstructive pulmonary disease | 21,930 | 41,217 | 87.9 | 94.9 | 63.9 | -32.6 |
| B.3.2 - Pneumoconiosis | 142 | 401 | 181.2 | 0.4 | 0.5 | 23.3 |
| B.3.2.1 - Silicosis | 93 | 237 | 153.4 | 0.3 | 0.3 | 16.7 |
| B.3.2.2 - Asbestosis | 9 | 45 | 379.2 | 0.0 | 0.1 | 113.2 |
| B.3.2.3 - Coal workers pneumoconiosis | 12 | 40 | 240.5 | 0.0 | 0.1 | 49.9 |
| B.3.2.4 - Other pneumoconiosis | 28 | 79 | 182.9 | 0.1 | 0.1 | 3.9 |
| B.3.3 - Asthma | 1,255 | 1,517 | 20.9 | 3.6 | 2.0 | -43.7 |
| B.3.4 - Interstitial lung disease and pulmonary sarcoidosis | 435 | 1,611 | 270.5 | 1.4 | 2.3 | 58.3 |
| B.3.5 - Other chronic respiratory diseases | 351 | 511 | 45.4 | 0.6 | 0.6 | 7.3 |
| **B.4 - Cirrhosis and other chronic liver diseases** | **17,000** | **27,441** | **61.4** | **40.3** | **30.4** | **-24.7** |
| B.4.1 - Cirrhosis and other chronic liver diseases due to hepatitis B | 2,945 | 4,441 | 50.8 | 7.7 | 5.1 | -33.2 |
| B.4.2 - Cirrhosis and other chronic liver diseases due to hepatitis C | 3,460 | 5,448 | 57.5 | 8.6 | 6.1 | -29.0 |
| B.4.3 - Cirrhosis and other chronic liver diseases due to alcohol use | 8,720 | 15,270 | 75.1 | 19.7 | 16.5 | -16.3 |
| B.4.4 - Cirrhosis and other chronic liver diseases due to other causes | 1,876 | 2,281 | 21.6 | 4.4 | 2.7 | -39.3 |
| **B.5 - Digestive diseases** | **10,110** | **17,268** | **70.8** | **31.7** | **23.2** | **-26.8** |
| B.5.1 - Peptic ulcer disease | 2,996 | 2,716 | -9.3 | 9.7 | 3.6 | -62.3 |
| B.5.2 - Gastritis and duodenitis | 191 | 396 | 107.1 | 0.6 | 0.5 | -13.2 |
| B.5.3 - Appendicitis | 512 | 728 | 42.3 | 1.2 | 0.9 | -25.4 |
| B.5.4 - Paralytic ileus and intestinal obstruction | 1,323 | 2,527 | 90.9 | 4.3 | 3.6 | -17.0 |
| B.5.5 - Inguinal, femoral, and abdominal hernia | 497 | 857 | 72.3 | 1.9 | 1.2 | -32.8 |
| B.5.6 - Inflammatory bowel disease | 229 | 456 | 98.9 | 0.6 | 0.6 | -10.4 |
| B.5.7 - Vascular intestinal disorders | 982 | 1,809 | 84.3 | 3.5 | 2.6 | -27.1 |
| B.5.8 - Gallbladder and biliary diseases | 912 | 2,281 | 150.2 | 3.3 | 3.3 | -0.7 |
| B.5.9 - Pancreatitis | 1,401 | 3,092 | 120.7 | 3.2 | 3.5 | 9.7 |
| B.5.10 - Other digestive diseases | 1,067 | 2,407 | 125.6 | 3.3 | 3.3 | -1.1 |
| **B.6 - Neurological disorders** | **8,838** | **27,769** | **214.2** | **47.6** | **46.6** | **-2.0** |
| B.6.1 - Alzheimer disease and other dementias | 6,639 | 22,644 | 241.1 | 42.6 | 40.0 | -6.2 |
| B.6.2 - Parkinson disease | 286 | 1,860 | 551.3 | 1.4 | 3.1 | 124.1 |
| B.6.3 - Epilepsy | 1,143 | 1,604 | 40.3 | 1.8 | 1.6 | -9.2 |
| B.6.4 - Multiple sclerosis | 63 | 157 | 149.7 | 0.2 | 0.2 | 11.2 |
| B.6.8 - Motor neuron disease | 190 | 591 | 211.6 | 0.4 | 0.7 | 55.1 |
| B.6.8 - Other neurological disorders | 519 | 914 | 76.2 | 1.1 | 1.0 | -7.5 |
| **B.7 - Mental and substance use disorders** | **4,375** | **9,409** | **115.0** | **8.3** | **9.5** | **14.5** |
| B.7.1 - Schizophrenia | 49 | 221 | 349.4 | 0.1 | 0.2 | 114.8 |
| B.7.2 - Alcohol use disorders | 3,909 | 8,095 | 107.1 | 7.5 | 8.1 | 9.0 |
| B.7.3 - Drug use disorders | 411 | 1,077 | 161.8 | 0.7 | 1.1 | 56.2 |
| B.7.3.1 - Opioid use disorders | 356 | 649 | 82.5 | 0.6 | 0.7 | 18.3 |
| B.7.3.2 - Cocaine use disorders | 11 | 175 | 1516.8 | 0.0 | 0.2 | 698.1 |
| B.7.3.3 - Amphetamine use disorders | 2 | 31 | 1536.5 | 0.0 | 0.0 | 725.0 |
| B.7.3.5 - Other drug use disorders | 43 | 222 | 414.9 | 0.1 | 0.2 | 104.3 |
| B.7.7 - Eating disorders | 6 | 16 | 183.0 | 0.0 | 0.0 | 83.9 |
| B.7.7.1 - Anorexia nervosa | 5 | 16 | 184.4 | 0.0 | 0.0 | 87.3 |
| B.7.7.2 - Bulimia nervosa | 0 | 1 | 161.5 | 0.0 | 0.0 | 40.1 |
| **B.8 - Diabetes, urogenital, blood, and endocrine diseases** | **21,699** | **57,517** | **165.1** | **70.8** | **80.0** | **13.0** |
| B.8.1 - Diabetes mellitus | 9,770 | 27,416 | 180.6 | 32.8 | 38.1 | 16.2 |
| B.8.2 - Acute glomerulonephritis | 125 | 32 | -74.3 | 0.2 | 0.0 | -81.1 |
| B.8.3 - Chronic kidney disease | 8,237 | 19,083 | 131.7 | 26.9 | 26.5 | -1.3 |
| B.8.3.1 - Chronic kidney disease due to diabetes mellitus | 3,934 | 11,327 | 187.9 | 13.3 | 15.3 | 14.9 |
| B.8.3.2 - Chronic kidney disease due to hypertension | 3,473 | 6,748 | 94.3 | 11.2 | 9.8 | -12.7 |
| B.8.3.3 - Chronic kidney disease due to glomerulonephritis | 779 | 905 | 16.2 | 2.2 | 1.3 | -40.8 |
| B.8.3.4 - Chronic kidney disease due to other causes | 52 | 103 | 98.0 | 0.2 | 0.2 | -5.6 |
| B.8.4 - Urinary diseases and male infertility | 1,830 | 5,913 | 223.1 | 6.9 | 9.1 | 30.7 |
| B.8.4.1 - Interstitial nephritis and urinary tract infections | 1,447 | 4,997 | 245.2 | 5.5 | 7.8 | 42.0 |
| B.8.4.2 - Urolithiasis | 46 | 135 | 190.9 | 0.1 | 0.2 | 23.1 |
| B.8.4.5 - Other urinary diseases | 336 | 781 | 132.2 | 1.3 | 1.1 | -16.6 |
| B.8.5 - Gynecological diseases | 0 | 0 | - | 0.0 | 0.0 | - |
| B.8.5.1 - Uterine fibroids | 0 | 0 | - | 0.0 | 0.0 | - |
| B.8.5.2 - Polycystic ovarian syndrome | 0 | 0 | - | 0.0 | 0.0 | - |
| B.8.5.4 - Endometriosis | 0 | 0 | - | 0.0 | 0.0 | - |
| B.8.5.5 - Genital prolapse | 0 | 0 | - | 0.0 | 0.0 | - |
| B.8.5.7 - Other gynecological diseases | 0 | 0 | - | 0.0 | 0.0 | - |
| B.8.6 - Hemoglobinopathies and hemolytic anemias | 765 | 1,062 | 38.7 | 1.8 | 1.3 | -28.0 |
| B.8.6.1 - Thalassemias | 79 | 59 | -25.3 | 0.1 | 0.1 | -22.0 |
| B.8.6.2 - Sickle cell disorders | 152 | 192 | 25.8 | 0.2 | 0.2 | -0.3 |
| B.8.6.3 - G6PD deficiency | 190 | 329 | 72.9 | 0.3 | 0.3 | 5.6 |
| B.8.6.4 - Other hemoglobinopathies and hemolytic anemias | 343 | 482 | 40.3 | 1.2 | 0.7 | -41.7 |
| B.8.7 - Endocrine, metabolic, blood, and immune disorders | 971 | 4,011 | 313.2 | 2.2 | 5.0 | 128.1 |
| **B.9 - Musculoskeletal disorders** | **414** | **944** | **127.8** | **1.1** | **1.2** | **5.7** |
| B.9.1 - Rheumatoid arthritis | 72 | 158 | 119.6 | 0.2 | 0.2 | -9.5 |
| B.9.5 - Other musculoskeletal disorders | 343 | 787 | 129.5 | 0.9 | 1.0 | 9.6 |
| **B.10 - Other non-communicable diseases** | **8,072** | **9,044** | **12.0** | **10.1** | **11.2** | **10.0** |
| B.10.1 - Congenital anomalies | 6,942 | 6,215 | -10.5 | 7.2 | 7.2 | 0.6 |
| B.10.1.1 - Neural tube defects | 590 | 344 | -41.7 | 0.6 | 0.4 | -31.5 |
| B.10.1.2 - Congenital heart anomalies | 3,747 | 2,623 | -30.0 | 3.9 | 3.0 | -22.1 |
| B.10.1.3 - Cleft lip and cleft palate | 24 | 14 | -43.3 | 0.0 | 0.0 | -32.5 |
| B.10.1.4 - Down syndrome | 190 | 254 | 33.7 | 0.2 | 0.3 | 40.1 |
| B.10.1.7 - Other chromosomal abnormalities | 43 | 150 | 248.6 | 0.0 | 0.2 | 295.4 |
| B.10.1.8 - Other congenital anomalies | 2,348 | 2,831 | 20.6 | 2.5 | 3.3 | 35.8 |
| B.10.2 - Skin and subcutaneous diseases | 1,013 | 2,681 | 164.6 | 2.8 | 3.7 | 32.2 |
| B.10.2.3 - Cellulitis | 102 | 299 | 193.4 | 0.3 | 0.4 | 55.5 |
| B.10.2.4 - Pyoderma | 650 | 1,631 | 151.0 | 1.6 | 2.2 | 38.3 |
| B.10.2.12 - Decubitus ulcer | 203 | 647 | 219.5 | 0.8 | 1.0 | 19.8 |
| B.10.2.13 - Other skin and subcutaneous diseases | 59 | 105 | 76.4 | 0.1 | 0.1 | -6.3 |
| B.10.5 - Sudden infant death syndrome | 117 | 148 | 26.6 | 0.1 | 0.2 | 50.1 |
| **C - Injuries** | **107,868** | **136,242** | **26.3** | **171.0** | **134.2** | **-21.5** |
| **C.1 - Transport injuries** | **37,825** | **43,996** | **16.3** | **60.3** | **42.9** | **-28.8** |
| C.1.1 - Road injuries | 37,400 | 42,084 | 12.5 | 59.6 | 41.1 | -31.1 |
| C.1.1.1 - Pedestrian road injuries | 18,608 | 16,577 | -10.9 | 31.6 | 17.1 | -45.8 |
| C.1.1.2 - Cyclist road injuries | 865 | 1,837 | 112.4 | 1.4 | 1.8 | 34.0 |
| C.1.1.3 - Motorcyclist road injuries | 5,037 | 11,617 | 130.7 | 7.0 | 10.5 | 51.6 |
| C.1.1.4 - Motor vehicle road injuries | 12,757 | 11,356 | -11.0 | 19.5 | 10.9 | -44.2 |
| C.1.1.5 - Other road injuries | 135 | 698 | 418.6 | 0.2 | 0.7 | 215.0 |
| C.1.2 - Other transport injuries | 424 | 1,912 | 350.9 | 0.7 | 1.8 | 181.1 |
| **C.2 - Unintentional injuries** | **25,235** | **25,801** | **2.2** | **45.2** | **29.6** | **-34.5** |
| C.2.1 - Falls | 5,029 | 9,442 | 87.7 | 13.6 | 12.2 | -10.2 |
| C.2.2 - Drowning | 8,461 | 5,887 | -30.4 | 11.0 | 5.7 | -48.1 |
| C.2.3 - Fire, heat, and hot substances | 1,677 | 1,177 | -29.8 | 3.1 | 1.4 | -55.0 |
| C.2.4 - Poisonings | 406 | 242 | -40.4 | 0.6 | 0.3 | -56.6 |
| C.2.5 - Exposure to mechanical forces | 2,965 | 2,251 | -24.1 | 4.4 | 2.3 | -49.1 |
| C.2.5.1 - Unintentional firearm injuries | 1,183 | 755 | -36.2 | 1.9 | 0.8 | -58.8 |
| C.2.5.2 - Unintentional suffocation | 418 | 166 | -60.2 | 0.4 | 0.2 | -59.4 |
| C.2.5.3 - Other exposure to mechanical forces | 1,364 | 1,330 | -2.5 | 2.1 | 1.3 | -38.5 |
| C.2.6 - Adverse effects of medical treatment | 1,369 | 1,514 | 10.6 | 3.1 | 1.9 | -39.2 |
| C.2.7 - Animal contact | 489 | 454 | -7.2 | 0.9 | 0.5 | -43.6 |
| C.2.7.1 - Venomous animal contact | 366 | 274 | -25.2 | 0.7 | 0.3 | -54.2 |
| C.2.7.2 - Non-venomous animal contact | 124 | 181 | 45.8 | 0.3 | 0.2 | -15.1 |
| C.2.8 - Foreign body | 2,251 | 1,922 | -14.6 | 4.2 | 2.4 | -42.7 |
| C.2.8.1 - Pulmonary aspiration and foreign body in airway | 1,949 | 1,691 | -13.2 | 3.5 | 2.1 | -40.0 |
| C.2.8.3 - Foreign body in other body part | 302 | 231 | -23.4 | 0.7 | 0.3 | -57.1 |
| C.2.9 - Other unintentional injuries | 2,226 | 2,688 | 20.8 | 3.3 | 2.7 | -18.4 |
| C.2.10 - Environmental heat and cold exposure | 361 | 223 | -38.3 | 0.9 | 0.3 | -72.5 |
| **C.3 - Self-harm and interpersonal violence** | **44,631** | **66,445** | **48.9** | **65.2** | **61.6** | **-5.5** |
| C.3.1 - Self-harm | 7,710 | 11,148 | 44.6 | 13.3 | 10.9 | -18.1 |
| C.3.2 - Interpersonal violence | 36,921 | 55,297 | 49.8 | 51.9 | 50.7 | -2.3 |
| C.3.2.1 - Assault by firearm | 21,165 | 39,700 | 87.6 | 29.1 | 36.1 | 23.9 |
| C.3.2.2 - Assault by sharp object | 6,382 | 9,301 | 45.7 | 9.4 | 8.6 | -8.1 |
| C.3.2.3 - Assault by other means | 9,374 | 6,296 | -32.8 | 13.5 | 6.1 | -54.9 |
| **C.4 - Forces of nature, war, and legal intervention** | **177** | **0** | **-100.0** | **0.3** | **0.0** | **-100.0** |
| C.4.1 - Exposure to forces of nature | 177 | 0 | -100.0 | 0.3 | 0.0 | -100.0 |
| C.4.2 - Collective violence and legal intervention | 0 | 0 | - | 0.0 | 0.0 | - |
|  |  |  |  |  |  |  |

| **Additional file 2: Table C- Number of deaths and age-standardized rates for 249 causes of death, female. Brazil, 1990 and 2015.** | | | | | | |
| --- | --- | --- | --- | --- | --- | --- |
|  |  |  |  |  |  |  |
| **Causes of death** | **Number of deaths** | | | **Age-standardized death rates (per 100,000)** | | |
|  | **1990** | **2015** | **% change*** | **1990** | **2015** | **% change*** |
| **All causes** | **377,201** | **584,525** | **55.0** | **869.6** | **609.6** | **-29.9** |
| **A - Communicable, maternal, neonatal, and nutritional diseases** | **103,505** | **70,687** | **-31.7** | **152.2** | **76.5** | **-49.7** |
| **A.1 - HIV/AIDS and tuberculosis** | **3,847** | **6,956** | **80.8** | **6.7** | **6.3** | **-7.1** |
| A.1.1 - Tuberculosis | 2,531 | 1,578 | -37.7 | 4.9 | 1.5 | -68.6 |
| A.1.2 - HIV/AIDS | 1,316 | 5,378 | 308.6 | 1.9 | 4.7 | 152.1 |
| A.1.2.1 - HIV/AIDS - Tuberculosis | 346 | 505 | 45.9 | 0.5 | 0.4 | -9.9 |
| A.1.2.2 - HIV/AIDS resulting in other diseases | 970 | 4,873 | 402.4 | 1.4 | 4.3 | 210.0 |
| **A.2 - Diarrhea, lower respiratory, and other common infectious diseases** | **53,731** | **41,672** | **-22.4** | **87.7** | **45.0** | **-48.7** |
| A.2.1 - Diarrheal diseases | 20,866 | 3,130 | -85.0 | 26.7 | 3.5 | -87.0 |
| A.2.2 - Intestinal infectious diseases | 65 | 28 | -57.3 | 0.1 | 0.0 | -69.9 |
| A.2.2.1 - Typhoid fever | 25 | 20 | -22.2 | 0.0 | 0.0 | -53.3 |
| A.2.2.2 - Paratyphoid fever | 0 | 1 | 253.7 | 0.0 | 0.0 | 126.5 |
| A.2.2.3 - Other intestinal infectious diseases | 40 | 8 | -80.9 | 0.1 | 0.0 | -84.3 |
| A.2.3 - Lower respiratory infections | 26,724 | 37,016 | 38.5 | 53.6 | 39.9 | -25.5 |
| A.2.4 - Upper respiratory infections | 20 | 19 | -5.5 | 0.0 | 0.0 | -49.2 |
| A.2.5 - Otitis media | 87 | 24 | -72.5 | 0.1 | 0.0 | -75.9 |
| A.2.6 - Meningitis | 3,464 | 1,135 | -67.2 | 4.1 | 1.2 | -70.8 |
| A.2.6.1 - Pneumococcal meningitis | 630 | 177 | -72.0 | 0.7 | 0.2 | -73.8 |
| A.2.6.2 - H influenzae type B meningitis | 441 | 40 | -90.9 | 0.5 | 0.0 | -90.5 |
| A.2.6.3 - Meningococcal meningitis | 1,144 | 402 | -64.9 | 1.3 | 0.4 | -67.9 |
| A.2.6.4 - Other meningitis | 1,248 | 516 | -58.6 | 1.6 | 0.5 | -65.6 |
| A.2.7 - Encephalitis | 328 | 205 | -37.4 | 0.4 | 0.2 | -50.9 |
| A.2.8 - Diphtheria | 13 | 1 | -89.3 | 0.0 | 0.0 | -89.8 |
| A.2.9 - Whooping cough | 1,046 | 47 | -95.6 | 1.1 | 0.1 | -94.7 |
| A.2.10 - Tetanus | 409 | 42 | -89.8 | 0.7 | 0.0 | -93.6 |
| A.2.11 - Measles | 548 | 2 | -99.7 | 0.6 | 0.0 | -99.7 |
| A.2.12 - Varicella and herpes zoster | 162 | 25 | -84.7 | 0.3 | 0.0 | -91.7 |
| **A.3 - Neglected tropical diseases and malaria** | **3,586** | **3,185** | **-11.2** | **7.8** | **3.2** | **-58.7** |
| A.3.1 - Malaria | 282 | 37 | -87.0 | 0.3 | 0.0 | -89.2 |
| A.3.2 - Chagas disease | 2,650 | 2,555 | -3.6 | 6.2 | 2.6 | -58.1 |
| A.3.3 - Leishmaniasis | 104 | 160 | 54.7 | 0.2 | 0.2 | 3.8 |
| A.3.3.1 - Visceral leishmaniasis | 104 | 160 | 54.7 | 0.2 | 0.2 | 3.8 |
| A.3.4 - African trypanosomiasis | 0 | 0 | - | 0.0 | 0.0 | - |
| A.3.5 - Schistosomiasis | 365 | 110 | -69.7 | 0.8 | 0.1 | -87.1 |
| A.3.6 - Cysticercosis | 64 | 15 | -76.2 | 0.1 | 0.0 | -87.9 |
| A.3.7 - Cystic echinococcosis | 7 | 3 | -62.6 | 0.0 | 0.0 | -81.2 |
| A.3.11 - Dengue | 15 | 243 | 1530.4 | 0.0 | 0.2 | 960.9 |
| A.3.12 - Yellow fever | 4 | 2 | -61.3 | 0.0 | 0.0 | -71.0 |
| A.3.13 - Rabies | 17 | 1 | -96.0 | 0.0 | 0.0 | -96.9 |
| A.3.14 - Intestinal nematode infections | 14 | 6 | -55.6 | 0.0 | 0.0 | -59.6 |
| A.3.14.1 - Ascariasis | 14 | 6 | -55.6 | 0.0 | 0.0 | -59.6 |
| A.3.17 - Ebola | 0 | 0 | - | 0.0 | 0.0 | - |
| A.3.19 - Other neglected tropical diseases | 64 | 53 | -17.7 | 0.1 | 0.1 | -30.1 |
| **A.4 - Maternal disorders** | **3,081** | **1,972** | **-36.0** | **3.9** | **1.7** | **-55.6** |
| A.4.1 - Maternal hemorrhage | 520 | 289 | -44.4 | 0.7 | 0.3 | -62.3 |
| A.4.2 - Maternal sepsis and other maternal infections | 443 | 237 | -46.6 | 0.6 | 0.2 | -63.2 |
| A.4.3 - Maternal hypertensive disorders | 737 | 403 | -45.4 | 0.9 | 0.4 | -60.7 |
| A.4.4 - Maternal obstructed labor and uterine rupture | 55 | 37 | -33.5 | 0.1 | 0.0 | -56.0 |
| A.4.5 - Maternal abortion, miscarriage, and ectopic pregnancy | 458 | 250 | -45.4 | 0.6 | 0.2 | -64.6 |
| A.4.6 - Indirect maternal deaths | 439 | 403 | -8.2 | 0.5 | 0.4 | -34.0 |
| A.4.7 - Late maternal deaths | 100 | 89 | -10.6 | 0.1 | 0.1 | -37.5 |
| A.4.8 - Maternal deaths aggravated by HIV/AIDS | 1 | 2 | 53.1 | 0.0 | 0.0 | 6.1 |
| A.4.9 - Other maternal disorders | 326 | 262 | -19.9 | 0.4 | 0.2 | -43.8 |
| **A.5 - Neonatal disorders** | **31,043** | **11,065** | **-64.4** | **32.7** | **14.0** | **-57.2** |
| A.5.1 - Neonatal preterm birth complications | 18,405 | 4,173 | -77.3 | 19.4 | 5.3 | -72.8 |
| A.5.2 - Neonatal encephalopathy due to birth asphyxia and trauma | 6,050 | 2,521 | -58.3 | 6.4 | 3.2 | -50.0 |
| A.5.3 - Neonatal sepsis and other neonatal infections | 4,351 | 2,347 | -46.1 | 4.6 | 3.0 | -35.4 |
| A.5.4 - Hemolytic disease and other neonatal jaundice | 409 | 77 | -81.1 | 0.4 | 0.1 | -77.3 |
| A.5.5 - Other neonatal disorders | 1,828 | 1,946 | 6.5 | 1.9 | 2.5 | 27.7 |
| **A.6 - Nutritional deficiencies** | **6,190** | **4,519** | **-27.0** | **10.6** | **4.9** | **-53.3** |
| A.6.1 - Protein-energy malnutrition | 5,822 | 4,161 | -28.5 | 9.9 | 4.6 | -54.0 |
| A.6.2 - Iodine deficiency | 16 | 12 | -26.3 | 0.0 | 0.0 | -55.5 |
| A.6.4 - Iron-deficiency anemia | 7 | 14 | 112.7 | 0.0 | 0.0 | 45.0 |
| A.6.5 - Other nutritional deficiencies | 345 | 331 | -3.8 | 0.6 | 0.4 | -44.9 |
| **A.7 - Other communicable, maternal, neonatal, and nutritional diseases** | **2,027** | **1,317** | **-35.0** | **2.7** | **1.3** | **-51.0** |
| A.7.1 - Sexually transmitted diseases excluding HIV | 1,184 | 308 | -74.0 | 1.3 | 0.3 | -76.1 |
| A.7.1.1 - Syphilis | 1,162 | 282 | -75.8 | 1.3 | 0.3 | -77.3 |
| A.7.1.2 - Chlamydial infection | 4 | 5 | 24.1 | 0.0 | 0.0 | -35.6 |
| A.7.1.3 - Gonococcal infection | 11 | 15 | 28.5 | 0.0 | 0.0 | -35.1 |
| A.7.1.6 - Other sexually transmitted diseases | 6 | 7 | 11.7 | 0.0 | 0.0 | -39.1 |
| A.7.2 - Hepatitis | 464 | 450 | -3.0 | 0.8 | 0.4 | -43.6 |
| A.7.2.1 - Acute hepatitis A | 127 | 46 | -63.5 | 0.1 | 0.0 | -66.0 |
| A.7.2.2 - Acute hepatitis B | 317 | 378 | 19.3 | 0.6 | 0.4 | -39.0 |
| A.7.2.3 - Acute hepatitis C | 20 | 26 | 26.3 | 0.0 | 0.0 | -36.5 |
| A.7.2.4 - Acute hepatitis E | 0 | 0 | - | 0.0 | 0.0 | - |
| A.7.4 - Other infectious diseases | 379 | 559 | 47.6 | 0.6 | 0.6 | -3.7 |
| **B - Non-communicable diseases** | **246,633** | **482,063** | **95.5** | **673.1** | **501.8** | **-25.4** |
| **B.1 - Neoplasms** | **48,397** | **107,365** | **121.8** | **115.4** | **106.8** | **-7.5** |
| B.1.1 - Lip and oral cavity cancer | 483 | 1,264 | 161.5 | 1.3 | 1.3 | -2.1 |
| B.1.2 - Nasopharynx cancer | 83 | 182 | 118.2 | 0.2 | 0.2 | 5.4 |
| B.1.3 - Other pharynx cancer | 225 | 476 | 111.4 | 0.6 | 0.5 | -16.0 |
| B.1.4 - Esophageal cancer | 1,248 | 2,373 | 90.2 | 3.3 | 2.4 | -26.8 |
| B.1.5 - Stomach cancer | 4,299 | 6,898 | 60.4 | 11.5 | 7.0 | -38.9 |
| B.1.6 - Colon and rectum cancer | 3,698 | 10,917 | 195.3 | 9.9 | 11.1 | 12.5 |
| B.1.7 - Liver cancer | 2,167 | 4,545 | 109.8 | 5.6 | 4.6 | -17.2 |
| B.1.7.1 - Liver cancer due to hepatitis B | 392 | 679 | 73.3 | 0.9 | 0.7 | -24.4 |
| B.1.7.2 - Liver cancer due to hepatitis C | 914 | 1,971 | 115.6 | 2.5 | 2.0 | -18.5 |
| B.1.7.3 - Liver cancer due to alcohol use | 374 | 1,006 | 168.9 | 1.0 | 1.0 | 5.5 |
| B.1.7.4 - Liver cancer due to other causes | 487 | 889 | 82.7 | 1.2 | 0.9 | -27.4 |
| B.1.8 - Gallbladder and biliary tract cancer | 2,123 | 3,382 | 59.3 | 5.6 | 3.5 | -38.8 |
| B.1.9 - Pancreatic cancer | 2,024 | 5,962 | 194.6 | 5.5 | 6.1 | 11.5 |
| B.1.10 - Larynx cancer | 302 | 610 | 101.9 | 0.8 | 0.6 | -19.6 |
| B.1.11 - Tracheal, bronchus, and lung cancer | 4,222 | 12,749 | 202.0 | 10.6 | 12.8 | 20.7 |
| B.1.12 - Malignant skin melanoma | 321 | 865 | 169.2 | 0.7 | 0.9 | 14.8 |
| B.1.13 - Non-melanoma skin cancer | 237 | 948 | 300.5 | 0.7 | 1.0 | 43.9 |
| B.1.13.1 - Non-melanoma skin cancer (squamous-cell carcinoma) | 237 | 948 | 300.5 | 0.7 | 1.0 | 43.9 |
| B.1.14 - Mesothelioma | 385 | 270 | -30.0 | 1.1 | 0.3 | -75.8 |
| B.1.15 - Breast cancer | 7,265 | 16,964 | 133.5 | 16.4 | 16.3 | -0.5 |
| B.1.16 - Cervical cancer | 6,592 | 10,027 | 52.1 | 14.5 | 9.6 | -33.9 |
| B.1.17 - Uterine cancer | 1,200 | 2,321 | 93.4 | 3.1 | 2.4 | -24.4 |
| B.1.18 - Ovarian cancer | 1,868 | 4,455 | 138.6 | 4.2 | 4.3 | 2.7 |
| B.1.19 - Prostate cancer | 0 | 0 | - | 0.0 | 0.0 | - |
| B.1.20 - Testicular cancer | 0 | 0 | - | 0.0 | 0.0 | - |
| B.1.21 - Kidney cancer | 508 | 1,353 | 166.2 | 1.1 | 1.4 | 22.2 |
| B.1.22 - Bladder cancer | 505 | 1,547 | 206.3 | 1.5 | 1.6 | 10.9 |
| B.1.23 - Brain and nervous system cancer | 1,158 | 4,538 | 291.8 | 2.2 | 4.4 | 104.9 |
| B.1.24 - Thyroid cancer | 334 | 557 | 67.0 | 0.9 | 0.6 | -34.9 |
| B.1.25 - Hodgkin lymphoma | 206 | 260 | 25.9 | 0.4 | 0.2 | -36.0 |
| B.1.26 - Non-Hodgkin lymphoma | 1,129 | 2,800 | 147.9 | 2.5 | 2.8 | 13.8 |
| B.1.27 - Multiple myeloma | 464 | 1,572 | 239.2 | 1.1 | 1.6 | 38.2 |
| B.1.28 - Leukemia | 2,334 | 4,054 | 73.7 | 4.2 | 4.1 | -3.2 |
| B.1.28.1.1 - Acute lymphoid leukemia | 701 | 818 | 16.6 | 0.9 | 0.8 | -8.9 |
| B.1.28.1.2 - Chronic lymphoid leukemia | 183 | 525 | 187.4 | 0.5 | 0.6 | 4.9 |
| B.1.28.2.1 - Acute myeloid leukemia | 1,073 | 2,266 | 111.2 | 1.9 | 2.2 | 15.0 |
| B.1.28.2.2 - Chronic myeloid leukemia | 376 | 444 | 18.0 | 0.8 | 0.4 | -45.4 |
| B.1.29 - Other neoplasms | 3,017 | 5,476 | 81.5 | 6.2 | 5.5 | -11.8 |
| **B.2 - Cardiovascular diseases** | **122,935** | **200,250** | **62.9** | **358.3** | **210.7** | **-41.2** |
| B.2.1 - Rheumatic heart disease | 1,391 | 1,568 | 12.7 | 2.5 | 1.5 | -40.3 |
| B.2.2 - Ischemic heart disease | 55,742 | 88,462 | 58.7 | 169.9 | 93.2 | -45.1 |
| B.2.3 - Cerebrovascular disease | 48,771 | 70,592 | 44.7 | 139.7 | 74.3 | -46.8 |
| B.2.3.1 - Ischemic stroke | 14,943 | 22,936 | 53.5 | 51.4 | 25.1 | -51.2 |
| B.2.3.2 - Hemorrhagic stroke | 33,829 | 47,655 | 40.9 | 88.3 | 49.2 | -44.3 |
| B.2.4 - Hypertensive heart disease | 5,967 | 13,215 | 121.5 | 17.3 | 14.0 | -19.2 |
| B.2.5 - Cardiomyopathy and myocarditis | 4,533 | 8,113 | 79.0 | 11.4 | 8.5 | -25.8 |
| B.2.6 - Atrial fibrillation and flutter | 544 | 2,070 | 280.3 | 2.2 | 2.3 | 4.3 |
| B.2.7 - Aortic aneurysm | 920 | 3,799 | 312.9 | 2.4 | 3.9 | 64.6 |
| B.2.8 - Peripheral vascular disease | 211 | 1,264 | 497.8 | 0.7 | 1.4 | 94.8 |
| B.2.9 - Endocarditis | 505 | 1,179 | 133.5 | 1.0 | 1.2 | 15.7 |
| B.2.10 - Other cardiovascular and circulatory diseases | 4,350 | 9,988 | 129.6 | 11.1 | 10.4 | -6.4 |
| **B.3 - Chronic respiratory diseases** | **17,158** | **34,396** | **100.5** | **49.5** | **36.5** | **-26.3** |
| B.3.1 - Chronic obstructive pulmonary disease | 14,782 | 29,593 | 100.2 | 44.5 | 31.5 | -29.2 |
| B.3.2 - Pneumoconiosis | 13 | 102 | 674.0 | 0.0 | 0.1 | 190.6 |
| B.3.2.1 - Silicosis | 3 | 23 | 576.5 | 0.0 | 0.0 | 135.4 |
| B.3.2.2 - Asbestosis | 3 | 23 | 735.8 | 0.0 | 0.0 | 271.9 |
| B.3.2.3 - Coal workers pneumoconiosis | 2 | 20 | 892.3 | 0.0 | 0.0 | 234.3 |
| B.3.2.4 - Other pneumoconiosis | 5 | 36 | 618.2 | 0.0 | 0.0 | 173.1 |
| B.3.3 - Asthma | 1,545 | 2,480 | 60.5 | 3.5 | 2.5 | -26.8 |
| B.3.4 - Interstitial lung disease and pulmonary sarcoidosis | 335 | 1,740 | 419.7 | 0.9 | 1.8 | 111.1 |
| B.3.5 - Other chronic respiratory diseases | 484 | 481 | -0.6 | 0.7 | 0.5 | -21.4 |
| **B.4 - Cirrhosis and other chronic liver diseases** | **5,782** | **9,166** | **58.5** | **13.2** | **9.1** | **-31.3** |
| B.4.1 - Cirrhosis and other chronic liver diseases due to hepatitis B | 1,201 | 1,848 | 53.9 | 3.0 | 1.9 | -37.3 |
| B.4.2 - Cirrhosis and other chronic liver diseases due to hepatitis C | 1,256 | 1,838 | 46.3 | 3.0 | 1.8 | -39.4 |
| B.4.3 - Cirrhosis and other chronic liver diseases due to alcohol use | 1,987 | 3,653 | 83.8 | 4.3 | 3.5 | -17.9 |
| B.4.4 - Cirrhosis and other chronic liver diseases due to other causes | 1,338 | 1,827 | 36.5 | 2.9 | 1.9 | -36.5 |
| **B.5 - Digestive diseases** | **7,602** | **15,999** | **110.5** | **20.4** | **16.6** | **-18.5** |
| B.5.1 - Peptic ulcer disease | 1,535 | 2,028 | 32.1 | 4.3 | 2.1 | -51.1 |
| B.5.2 - Gastritis and duodenitis | 136 | 297 | 118.8 | 0.4 | 0.3 | -18.1 |
| B.5.3 - Appendicitis | 359 | 543 | 51.1 | 0.7 | 0.5 | -23.4 |
| B.5.4 - Paralytic ileus and intestinal obstruction | 1,237 | 2,622 | 112.0 | 3.4 | 2.8 | -17.9 |
| B.5.5 - Inguinal, femoral, and abdominal hernia | 349 | 749 | 114.3 | 1.0 | 0.8 | -20.3 |
| B.5.6 - Inflammatory bowel disease | 211 | 479 | 126.8 | 0.5 | 0.5 | 0.2 |
| B.5.7 - Vascular intestinal disorders | 910 | 2,000 | 119.8 | 2.7 | 2.1 | -21.1 |
| B.5.8 - Gallbladder and biliary diseases | 1,354 | 3,262 | 141.0 | 3.7 | 3.4 | -8.3 |
| B.5.9 - Pancreatitis | 656 | 1,637 | 149.5 | 1.5 | 1.6 | 6.9 |
| B.5.10 - Other digestive diseases | 854 | 2,381 | 178.7 | 2.2 | 2.5 | 11.2 |
| **B.6 - Neurological disorders** | **10,876** | **35,627** | **227.6** | **40.4** | **39.1** | **-3.2** |
| B.6.1 - Alzheimer disease and other dementias | 9,373 | 31,877 | 240.1 | 37.5 | 35.3 | -5.8 |
| B.6.2 - Parkinson disease | 224 | 1,477 | 558.4 | 0.7 | 1.6 | 116.2 |
| B.6.3 - Epilepsy | 673 | 860 | 27.7 | 1.0 | 0.8 | -14.6 |
| B.6.4 - Multiple sclerosis | 82 | 232 | 182.2 | 0.2 | 0.2 | 21.6 |
| B.6.8 - Motor neuron disease | 136 | 472 | 246.5 | 0.3 | 0.5 | 64.7 |
| B.6.8 - Other neurological disorders | 387 | 709 | 83.1 | 0.8 | 0.7 | -9.3 |
| **B.7 - Mental and substance use disorders** | **704** | **1,628** | **131.2** | **1.3** | **1.5** | **17.5** |
| B.7.1 - Schizophrenia | 37 | 160 | 325.8 | 0.1 | 0.2 | 94.4 |
| B.7.2 - Alcohol use disorders | 522 | 994 | 90.3 | 0.9 | 0.9 | -3.4 |
| B.7.3 - Drug use disorders | 135 | 457 | 239.0 | 0.3 | 0.4 | 71.6 |
| B.7.3.1 - Opioid use disorders | 103 | 265 | 158.2 | 0.2 | 0.3 | 34.9 |
| B.7.3.2 - Cocaine use disorders | 7 | 60 | 796.4 | 0.0 | 0.1 | 327.4 |
| B.7.3.3 - Amphetamine use disorders | 0 | 14 | 3754.7 | 0.0 | 0.0 | 1741.8 |
| B.7.3.5 - Other drug use disorders | 25 | 119 | 370.1 | 0.1 | 0.1 | 116.0 |
| B.7.7 - Eating disorders | 10 | 17 | 78.7 | 0.0 | 0.0 | 21.5 |
| B.7.7.1 - Anorexia nervosa | 9 | 16 | 69.9 | 0.0 | 0.0 | 16.9 |
| B.7.7.2 - Bulimia nervosa | 0 | 2 | 275.4 | 0.0 | 0.0 | 107.9 |
| **B.8 - Diabetes, urogenital, blood, and endocrine diseases** | **24,223** | **66,549** | **174.7** | **62.6** | **69.3** | **10.7** |
| B.8.1 - Diabetes mellitus | 14,032 | 35,049 | 149.8 | 38.0 | 36.7 | -3.4 |
| B.8.2 - Acute glomerulonephritis | 121 | 34 | -71.7 | 0.2 | 0.0 | -79.9 |
| B.8.3 - Chronic kidney disease | 6,551 | 17,254 | 163.4 | 16.2 | 17.8 | 9.9 |
| B.8.3.1 - Chronic kidney disease due to diabetes mellitus | 3,058 | 10,192 | 233.3 | 7.8 | 10.4 | 34.2 |
| B.8.3.2 - Chronic kidney disease due to hypertension | 2,695 | 6,185 | 129.5 | 6.7 | 6.4 | -3.4 |
| B.8.3.3 - Chronic kidney disease due to glomerulonephritis | 762 | 800 | 4.9 | 1.7 | 0.8 | -49.9 |
| B.8.3.4 - Chronic kidney disease due to other causes | 36 | 78 | 116.1 | 0.1 | 0.1 | 3.4 |
| B.8.4 - Urinary diseases and male infertility | 1,656 | 8,142 | 391.6 | 4.4 | 8.7 | 96.2 |
| B.8.4.1 - Interstitial nephritis and urinary tract infections | 1,539 | 7,666 | 398.2 | 4.2 | 8.2 | 96.7 |
| B.8.4.2 - Urolithiasis | 44 | 182 | 316.6 | 0.1 | 0.2 | 83.5 |
| B.8.4.5 - Other urinary diseases | 74 | 293 | 298.0 | 0.2 | 0.3 | 92.3 |
| B.8.5 - Gynecological diseases | 86 | 170 | 98.1 | 0.2 | 0.2 | -5.1 |
| B.8.5.1 - Uterine fibroids | 21 | 52 | 146.0 | 0.0 | 0.0 | 14.0 |
| B.8.5.2 - Polycystic ovarian syndrome | 3 | 1 | -63.7 | 0.0 | 0.0 | -82.3 |
| B.8.5.4 - Endometriosis | 2 | 8 | 247.4 | 0.0 | 0.0 | 101.5 |
| B.8.5.5 - Genital prolapse | 10 | 29 | 181.0 | 0.0 | 0.0 | 2.1 |
| B.8.5.7 - Other gynecological diseases | 49 | 79 | 62.9 | 0.1 | 0.1 | -16.0 |
| B.8.6 - Hemoglobinopathies and hemolytic anemias | 724 | 1,060 | 46.5 | 1.5 | 1.1 | -27.8 |
| B.8.6.1 - Thalassemias | 67 | 56 | -17.0 | 0.1 | 0.1 | -18.2 |
| B.8.6.2 - Sickle cell disorders | 149 | 227 | 53.0 | 0.2 | 0.2 | 10.9 |
| B.8.6.3 - G6PD deficiency | 92 | 169 | 83.1 | 0.1 | 0.2 | 14.5 |
| B.8.6.4 - Other hemoglobinopathies and hemolytic anemias | 416 | 608 | 46.3 | 1.1 | 0.6 | -40.8 |
| B.8.7 - Endocrine, metabolic, blood, and immune disorders | 1,053 | 4,839 | 359.6 | 2.1 | 4.9 | 127.4 |
| **B.9 - Musculoskeletal disorders** | **1,039** | **2,608** | **151.0** | **2.0** | **2.5** | **26.1** |
| B.9.1 - Rheumatoid arthritis | 181 | 434 | 139.8 | 0.5 | 0.4 | -5.5 |
| B.9.5 - Other musculoskeletal disorders | 858 | 2,174 | 153.4 | 1.5 | 2.1 | 35.7 |
| **B.10 - Other non-communicable diseases** | **7,916** | **8,475** | **7.1** | **9.9** | **9.7** | **-2.0** |
| B.10.1 - Congenital anomalies | 6,841 | 5,280 | -22.8 | 7.3 | 6.3 | -14.0 |
| B.10.1.1 - Neural tube defects | 830 | 370 | -55.4 | 0.9 | 0.5 | -47.3 |
| B.10.1.2 - Congenital heart anomalies | 3,680 | 2,215 | -39.8 | 3.9 | 2.6 | -34.2 |
| B.10.1.3 - Cleft lip and cleft palate | 25 | 13 | -50.5 | 0.0 | 0.0 | -40.8 |
| B.10.1.4 - Down syndrome | 210 | 204 | -2.8 | 0.2 | 0.2 | -0.7 |
| B.10.1.7 - Other chromosomal abnormalities | 65 | 194 | 196.9 | 0.1 | 0.2 | 242.1 |
| B.10.1.8 - Other congenital anomalies | 2,030 | 2,284 | 12.5 | 2.2 | 2.8 | 26.6 |
| B.10.2 - Skin and subcutaneous diseases | 988 | 3,085 | 212.2 | 2.5 | 3.3 | 31.0 |
| B.10.2.3 - Cellulitis | 77 | 333 | 330.7 | 0.2 | 0.3 | 102.0 |
| B.10.2.4 - Pyoderma | 579 | 1,672 | 188.7 | 1.3 | 1.8 | 35.3 |
| B.10.2.12 - Decubitus ulcer | 262 | 944 | 260.0 | 0.9 | 1.0 | 16.5 |
| B.10.2.13 - Other skin and subcutaneous diseases | 69 | 135 | 95.8 | 0.1 | 0.1 | -4.9 |
| B.10.5 - Sudden infant death syndrome | 87 | 110 | 26.8 | 0.1 | 0.1 | 50.8 |
| **C - Injuries** | **27,063** | **31,776** | **17.4** | **44.4** | **31.2** | **-29.6** |
| **C.1 - Transport injuries** | **10,794** | **10,605** | **-1.8** | **16.3** | **10.1** | **-38.3** |
| C.1.1 - Road injuries | 10,659 | 10,242 | -3.9 | 16.1 | 9.7 | -39.7 |
| C.1.1.1 - Pedestrian road injuries | 6,527 | 4,867 | -25.4 | 10.0 | 4.7 | -53.0 |
| C.1.1.2 - Cyclist road injuries | 147 | 311 | 111.9 | 0.2 | 0.3 | 42.4 |
| C.1.1.3 - Motorcyclist road injuries | 781 | 1,558 | 99.5 | 1.0 | 1.4 | 36.3 |
| C.1.1.4 - Motor vehicle road injuries | 3,155 | 3,411 | 8.1 | 4.8 | 3.2 | -32.8 |
| C.1.1.5 - Other road injuries | 50 | 96 | 91.8 | 0.1 | 0.1 | 28.8 |
| C.1.2 - Other transport injuries | 134 | 363 | 169.9 | 0.2 | 0.3 | 77.2 |
| **C.2 - Unintentional injuries** | **9,740** | **12,343** | **26.7** | **18.6** | **13.1** | **-29.8** |
| C.2.1 - Falls | 2,192 | 6,216 | 183.6 | 6.5 | 6.7 | 3.2 |
| C.2.2 - Drowning | 1,827 | 969 | -47.0 | 2.2 | 1.0 | -55.1 |
| C.2.3 - Fire, heat, and hot substances | 1,262 | 812 | -35.6 | 2.2 | 0.8 | -61.5 |
| C.2.4 - Poisonings | 251 | 196 | -21.6 | 0.4 | 0.2 | -45.8 |
| C.2.5 - Exposure to mechanical forces | 911 | 556 | -39.0 | 1.4 | 0.6 | -58.3 |
| C.2.5.1 - Unintentional firearm injuries | 304 | 185 | -39.0 | 0.6 | 0.2 | -67.9 |
| C.2.5.2 - Unintentional suffocation | 242 | 96 | -60.2 | 0.3 | 0.1 | -58.7 |
| C.2.5.3 - Other exposure to mechanical forces | 366 | 275 | -24.9 | 0.5 | 0.3 | -47.3 |
| C.2.6 - Adverse effects of medical treatment | 1,031 | 1,327 | 28.7 | 2.1 | 1.4 | -36.5 |
| C.2.7 - Animal contact | 163 | 160 | -1.8 | 0.2 | 0.2 | -35.2 |
| C.2.7.1 - Venomous animal contact | 129 | 114 | -11.9 | 0.2 | 0.1 | -42.9 |
| C.2.7.2 - Non-venomous animal contact | 34 | 47 | 36.7 | 0.0 | 0.0 | -3.5 |
| C.2.8 - Foreign body | 1,311 | 1,472 | 12.3 | 2.3 | 1.6 | -29.1 |
| C.2.8.1 - Pulmonary aspiration and foreign body in airway | 1,189 | 1,322 | 11.2 | 2.1 | 1.5 | -30.2 |
| C.2.8.3 - Foreign body in other body part | 122 | 150 | 23.2 | 0.2 | 0.2 | -17.1 |
| C.2.9 - Other unintentional injuries | 577 | 534 | -7.4 | 0.9 | 0.5 | -37.7 |
| C.2.10 - Environmental heat and cold exposure | 215 | 100 | -53.5 | 0.5 | 0.1 | -80.0 |
| **C.3 - Self-harm and interpersonal violence** | **6,417** | **8,828** | **37.6** | **9.3** | **8.1** | **-12.6** |
| C.3.1 - Self-harm | 2,172 | 2,888 | 33.0 | 3.4 | 2.6 | -23.0 |
| C.3.2 - Interpersonal violence | 4,245 | 5,940 | 39.9 | 5.9 | 5.5 | -6.6 |
| C.3.2.1 - Assault by firearm | 1,857 | 3,044 | 63.9 | 2.5 | 2.8 | 10.9 |
| C.3.2.2 - Assault by sharp object | 969 | 1,573 | 62.3 | 1.4 | 1.4 | 4.2 |
| C.3.2.3 - Assault by other means | 1,418 | 1,322 | -6.8 | 2.0 | 1.3 | -36.3 |
| **C.4 - Forces of nature, war, and legal intervention** | **113** | **0** | **-100.0** | **0.2** | **0.0** | **-100.0** |
| C.4.1 - Exposure to forces of nature | 113 | 0 | -100.0 | 0.2 | 0.0 | -100.0 |
| C.4.2 - Collective violence and legal intervention | 0 | 0 | - | 0.0 | 0.0 | - |
|  |  |  |  |  |  |  |
