# Supplementary material for: Cause-specific mortality for 249 causes in Brazil and states during 1990–2015: a systematic analysis for the global burden of disease study 2015
Source: Popul Health Metr. 2017 Nov 22;15:39. doi: 10.1186/s12963-017-0156-y (PMC5700707; doi:10.1186/s12963-017-0156-y)
Supplement: Supplementary file 6 — Age specific mortality rates (50–69 years and 70 and over) for urinary diseases and male infertility, both sexes, Brazil, 1990 to 2015. mortality rates for age groups 50–69 years and 70 and over for urinary diseases and male infertility, both sexes, Brazil, 1990 to 2015. (PDF 72 kb) [file 12963_2017_156_MOESM6_ESM.pdf]

These numbers are under embargo by the Lancet and may not be circulated.

Single | Explore | Compare ▼

Shared Settings

Use basic settings

Display: Cause | Risk | Etiology | Impairment

Cause: B.8.4 Urinary diseases and ...

Metric: Deaths

Location: Brazil

Sex: Male | Female | Both

Units: # | Rate | %

Uncertainty: On

Top chart Settings

Age: 50-69 years

Bottom chart Settings

Age: 70+ years

Take tour ▶

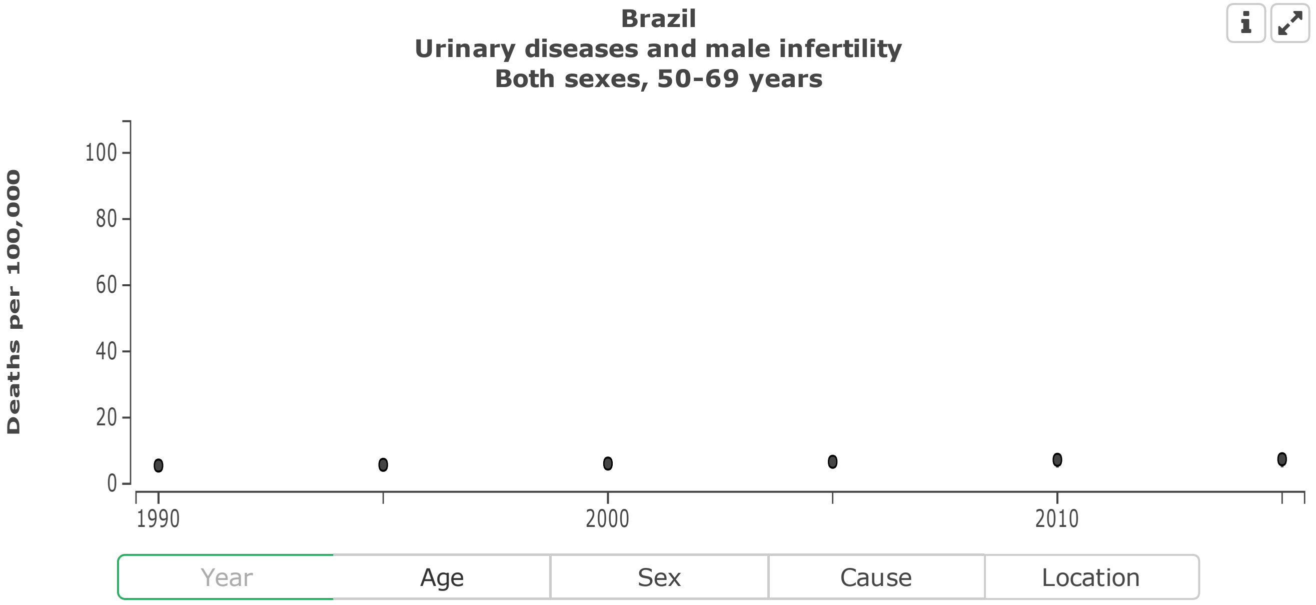

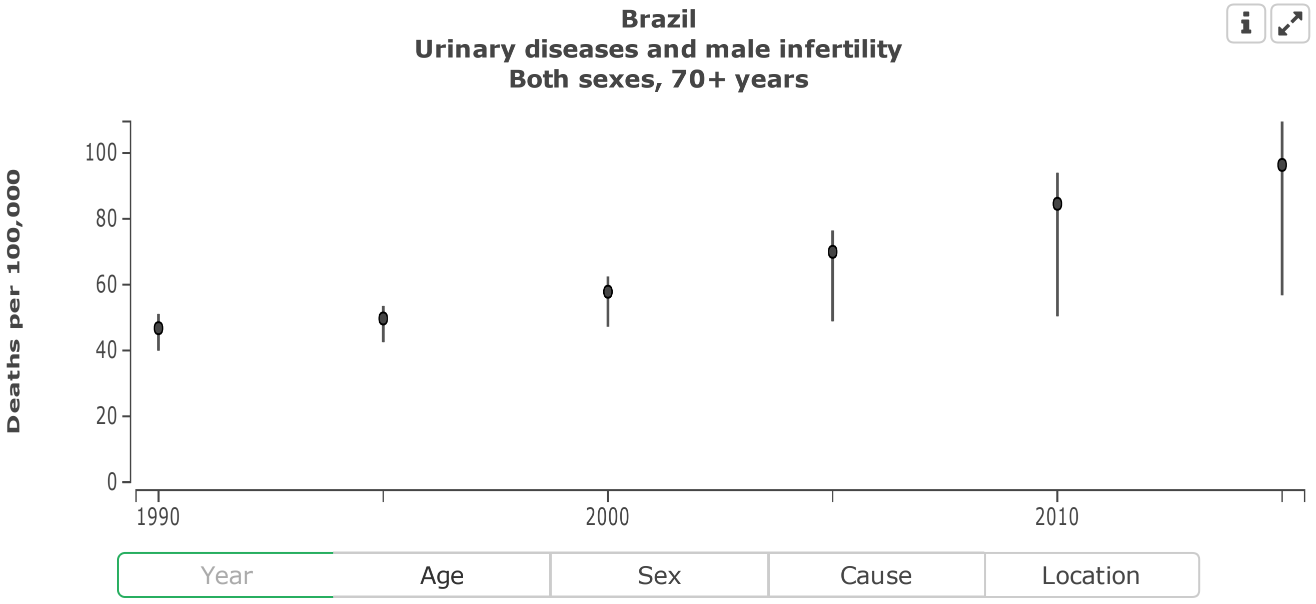

 © 2015 University of Washington
