# Supplementary material for: Cause-specific mortality for 249 causes in Brazil and states during 1990–2015: a systematic analysis for the global burden of disease study 2015
Source: Popul Health Metr. 2017 Nov 22;15:39. doi: 10.1186/s12963-017-0156-y (PMC5700707; doi:10.1186/s12963-017-0156-y)
Supplement: Supplementary file 7 — Trends in under-5 mortality rates due to lower respiratory infections compared to 70 years and over mortality rates in Brazil from 1990 to 2015. Mortality rates (per 100,000) due to lower respiratory infections from 1990 to 2015 among children under 5 years compared to the elderly (70 years and over) in Brazil from 1990 to 2015. (PDF 71 kb) [file 12963_2017_156_MOESM7_ESM.pdf]

GBD Compare | Viz Hub

These numbers are under embargo by the Lancet and may not be circulated.

Single | Explore | Compare ▼

Shared Settings

Use basic settings

Display: Cause | Risk | Etiology | Impairment

Cause: A.2.3 Lower respiratory infec...

Metric: Deaths

Location: Brazil

Sex: Male | Female | Both

Units: # | Rate | %

Uncertainty: On

Top chart Settings

Age: Under 5 years

Bottom chart Settings

Age: 70+ years

Take tour ▶

Brazil
Lower respiratory infections
Both sexes, Under 5 years

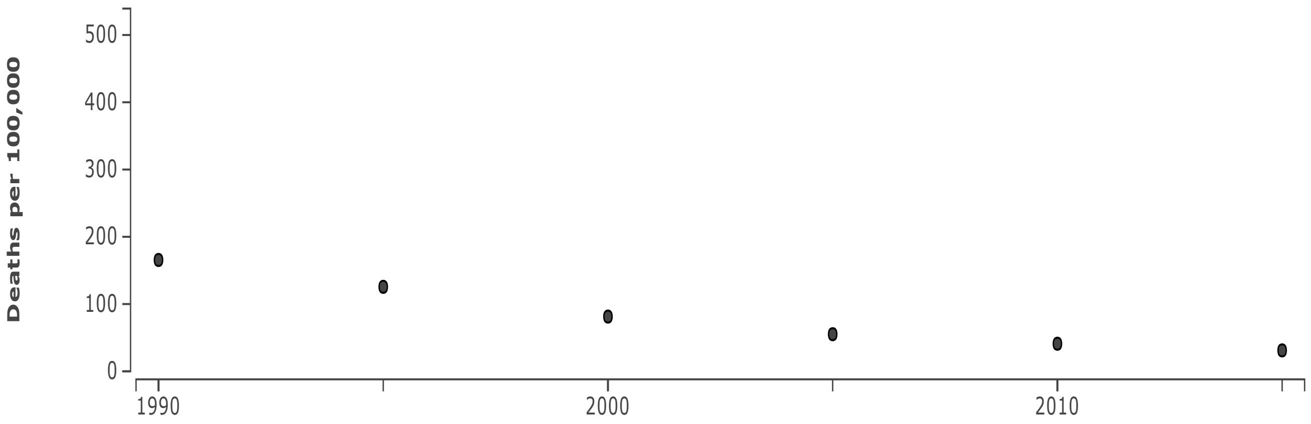

Year | Age | Sex | Cause | Location

Brazil
Lower respiratory infections
Both sexes, 70+ years

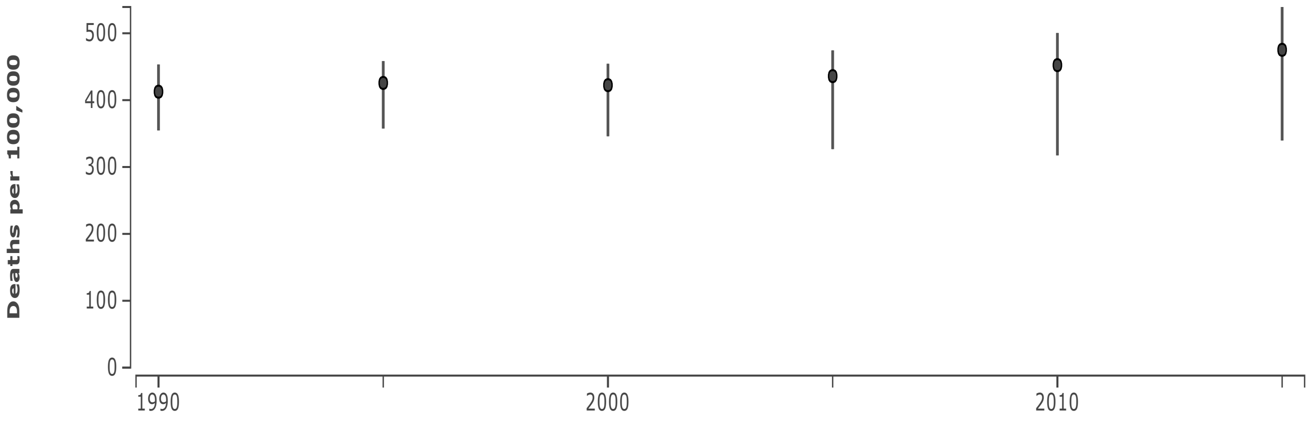

Year | Age | Sex | Cause | Location

 © 2015 University of Washington
